# Mapping human brain charts cross-sectionally and longitudinally

**DOI:** 10.1073/pnas.2216798120

**Published:** 2023-05-08

**Authors:** Maria A. Di Biase, Ye Ella Tian, Richard A. I. Bethlehem, Jakob Seidlitz, Aaron. F. Alexander-Bloch, B.T. Thomas Yeo, Andrew Zalesky

**Affiliations:** ^a^Melbourne Neuropsychiatry Centre, Department of Psychiatry, Melbourne Medical School, The University of Melbourne, Melbourne, VIC 3000, Australia; ^b^Department of Anatomy and Physiology, School of Biomedical Sciences, The University of Melbourne, Melbourne, VIC 3000, Australia; ^c^Department of Psychiatry, Brigham and Women's Hospital, Harvard Medical School, Boston, MA 02145; ^d^Department of Psychology, University of Cambridge, Cambridge CB2 3EB, United Kingdom; ^e^Department of Child and Adolescent Psychiatry and Behavioral Science, The Children’s Hospital of, Philadelphia, PA 19104; ^f^Lifespan Brain Institute, The Children’s Hospital of Philadelphia and Penn Medicine, Philadelphia, PA 19104; ^g^Department of Psychiatry, University of Pennsylvania, Philadelphia, PA 19104; ^h^Department of Electrical and Computer Engineering, National University of Singapore, Singapore City 119077, Singapore; ^i^Centre for Sleep & Cognition & Centre for Translational Magnetic Resonance Research, Yong Loo Lin School of Medicine, Singapore 119077, Singapore; ^j^N.1 Institute for Health & Institute for Digital Medicine, National University of Singapore, Singapore 119077, Singapore; ^k^Integrative Sciences and Engineering Programme, National University of Singapore, Singapore 119077, Singapore; ^l^Athinoula A. Martinos Center for Biomedical Imaging, Massachusetts General Hospital, Charlestown, MA 02129; ^m^Department of Biomedical Engineering, Faculty of Engineering & Information Technology, The University of Melbourne, Melbourne, VIC 3000, Australia

**Keywords:** normative models, cross-sectional, longitudinal, individual prediction, brain trajectory

## Abstract

Brain scans acquired across large, age-diverse cohorts have facilitated recent progress in establishing normative brain aging charts. Here, we ask the critical question of whether cross-sectional estimates of age-related brain trajectories resemble those directly measured from longitudinal data. We show that age-related brain changes inferred from cross-sectionally mapped brain charts can substantially underestimate actual changes measured longitudinally. We further find that brain aging trajectories vary markedly between individuals and are difficult to predict with population-level age trends estimated cross-sectionally. Prediction errors relate modestly to neuroimaging confounds and lifestyle factors. Our findings provide explicit evidence for the importance of longitudinal measurements in ascertaining brain development and aging trajectories.

## Significance

Charting how the brain develops is key to understanding abnormal brain changes in common psychiatric and neurological disorders. Pooling brain scans from large cohorts of individuals at a specific point in time—i.e., cross-sectionally—has allowed researchers to indirectly infer dynamic brain changes across the human lifespan. However, it is unknown whether this inference is accurate—do brain growth charts estimated from cross-sectional snapshots accurately mirror true brain changes observed in the same individuals scanned at multiple timepoints? Here, we demonstrate that brain charts inferred from cross-sectional data underestimate brain changes directly observed in longitudinal data. As we endeavor to accurately map human brain development, we must also incorporate longitudinal measurements of the brain.

Understanding brain aging is a fundamental challenge in neuroscience. An increased willingness to share neuroimaging datasets and the fruition of large-scale biobank initiatives have facilitated recent mapping of the world’s first normative brain reference charts spanning the entire human lifespan (1). Due to the time and cost of acquiring prospective data, brain aging trajectories are typically inferred from cross-sectional analyses, adopting age as a proxy for time—i.e., pseudolongitudinal designs. While cross-sectional designs afford well-powered and age-diverse insights, resulting estimates of age-related change may differ from observed longitudinal change (2).

Brain charts have informed the temporal patterning of macroscale brain aging, which broadly coincides with the timing of microcircuit development and decay. For example, volumetric gray matter reductions in the aging brain coincide with synaptic atrophy and dendritic regression (1). However, longitudinal brain phenotype trajectories—mapped from the same individuals at multiple timepoints—have revealed discrepant age-related trends, both in terms of regional development and in estimates of peak maturation (3, 4). These discrepancies may cast doubt on the validity of cross-sectionally derived age-related trends and undermine their potential for characterizing deviation patterns in clinical populations (5–7), subtyping (8), and biological classification (6, 9). Direct comparison in the same individuals may help to clarify whether cross-sectional estimates of age-related brain trajectories resemble those directly measured from longitudinal data.

Another major challenge presented by cross-sectional normative models is distinguishing age-related variability from nonage-related determinants of change. Different constellations of characteristics attributable to individuals, such as neuroimaging confounds, interscan interval, and lifestyle factors are known sources of nonage-related variability (10–13). Such variability may explain why person-specific rates of change in common MRI measures (e.g., cortical volume and thickness) ascertained directly from longitudinal measurements can often depart from group-level age-related brain trends—e.g., upward instead of downward slopes in gray matter volume (GMV) with advancing age (14). In turn, individualized brain trajectories may not mirror group-level trends. High technical/noise and/or biological variability ultimately pose challenges to clinical applications of brain age charts for individual-level prediction (15, 16), such as to predict atrophy rates in neurology cohorts or to understand deviant brain development and decline.

This investigation was motivated by the recent developments in mapping human brain charts (1, 17–20) and aimed to build on existing efforts to reconcile individual trajectories of brain change with cross-sectional-level inference (4). Here, we critically examine brain aging trajectories inferred from normative models of multimodal cross-sectional MRI data. Utilizing two independent datasets, reflecting aging and developmental cohorts, respectively, we test whether cross-sectionally inferred age-related trends recapitulate trajectories derived from longitudinal data and whether individual trajectories can be predicted using group-level rates of change inferred from cross-sectional normative models. This knowledge can inform future efforts to establish lifespan brain reference charts (1) and normative models of brain function (6).

## Results

**Fig. 1.**
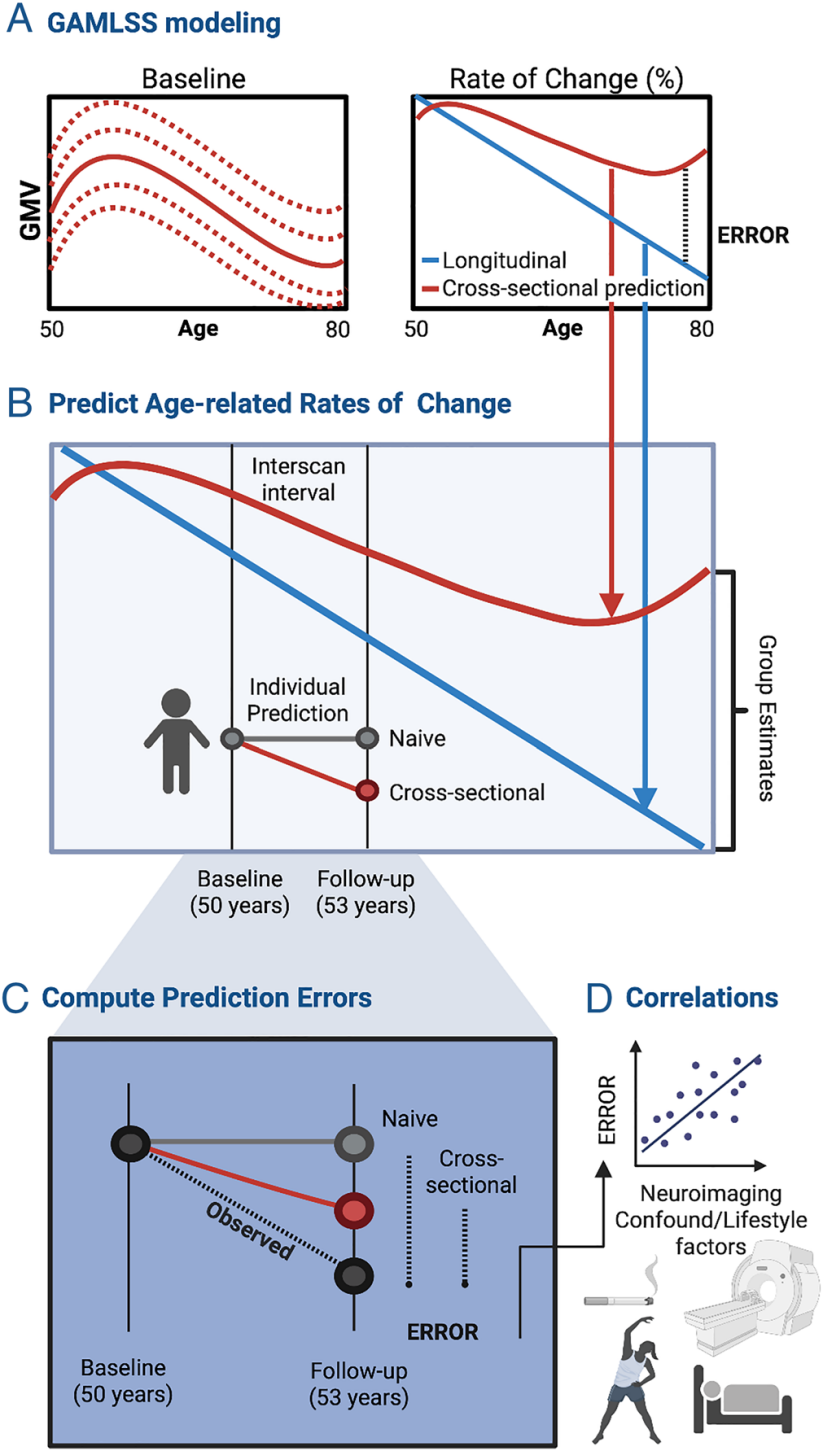
Study design. (*A*) GAMLSS frameworks established normative reference ranges of variation in cross-sectional baseline MRI measures (GMV, CTh, SA, and FA). (*B*) Numerical differentiation of the fitted cross-sectionally derived normative curves yielded group-level rates of change in MRI measures (red line), which were compared to rates of change directly ascertained from longitudinal measurements (blue line). Group-level estimates of rates of change were used to predict individual phenotype measurements at follow-up in unseen subjects. Specifically, individual trajectories were predicted from i) naive models assuming no change over time (i.e., equivalent baseline and follow-up values); ii) rates of change derived from cross-sectional data (50-th percentile); and rates of change derived from cross-sectional data using each individual’s percentile at baseline (*SI Appendix*, Fig. S5). (*C*) Errors in predicting individualized brain trajectories (i.e., mean absolute errors) were examined for (*D*) associations with demographic characteristics, neuroimaging confounds, and lifestyle variables across four domains: alcohol consumption, physical activity, sleep, and tobacco smoking. Abbreviations: gray matter volume (GMV); cortical thickness (CTh); surface area (SA); fractional anisotropy (FA); Cross (cross-sectional).

We first asked whether age-related rates of change apparent in normative brain aging charts accurately reflect rates of change measured longitudinally. To this end, brain MRI data were utilized from two cohorts (*SI Appendix*,Table S1): i) an aging cohort (age range = 47 to 80 y at baseline) comprising UK Biobank (UKB) (21) individuals with baseline and follow-up MRI data (N ranges from 2,752 to 2,832 with 48 to 49% females across the phenotypes), and ii) a developmental cohort (age range = 9 to 11 y at baseline) comprising Adolescent Brain Cognitive Development (ABCD) (22) individuals (N ranges from 6,537 to 7,480 with 54% females across the phenotypes) with available baseline and follow-up MRI. Results pertaining to the ABCD cohort are presented in the *SI Appendix*, Fig. 1 presents a methodological overview.

### Normative Models.

**Fig. 2.**
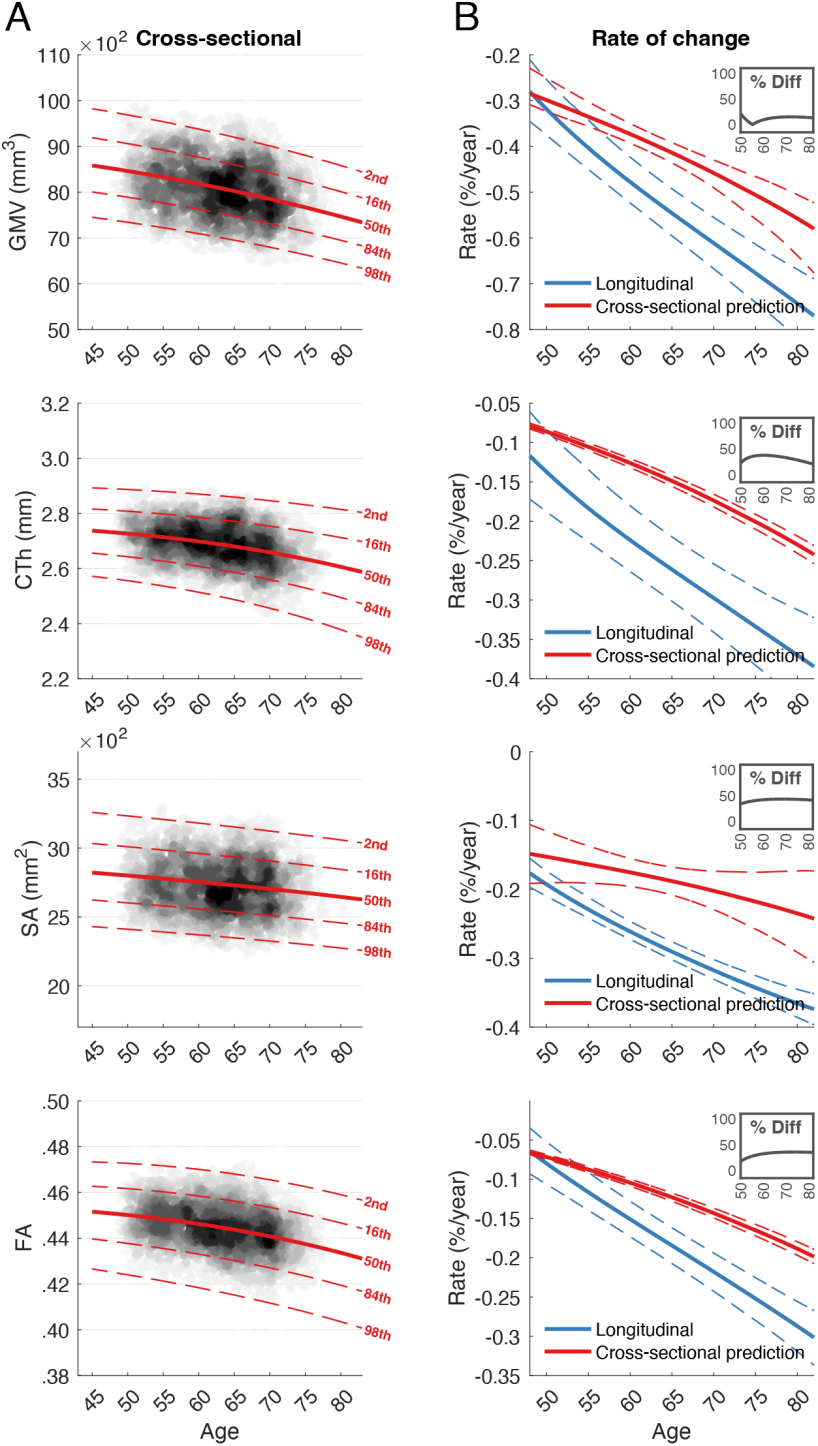
Normative models of brain aging. Normative centile reference ranges for (*A*) cross-sectionally measured whole-brain GMV, CTh, SA, and FA and (*B*) rates of change for each phenotype, shown alongside 25% and 75% CIs (dashed lines), generated with bootstrapping (500 samples). Rates of change were estimated i) directly from longitudinally measured phenotypes (blue) and ii) by differentiating the median centile curves (red). Insets show the percentage difference between cross-sectional and longitudinal rates of change. Abbreviations: gray matter volume (GMV); cortical thickness (CTh); surface area (SA); fractional anisotropy (FA); diff (difference).

Generalized Additive Models for Location, Scale and Shape (GAMLSS) frameworks (23) (stratified by sex and site) were used to establish normative reference ranges as a function of age for i) whole-brain cross-sectional GMV, cortical thickness (CTh), surface area (SA), and fractional anisotropy (FA) at baseline (Fig. 2*A*) and ii) annualized rates of change, estimated longitudinally (follow-up minus baseline divided by the interscan interval), for each of these four phenotypes (Fig. 2*B*, blue curves). Numerical differentiation (i.e., first derivative) of the fitted normative curves in Fig. 2*A* yielded cross-sectional group-level estimates of rates of change (Fig. 2*B*, red curves). This enabled the comparison of cross-sectionally inferred and longitudinally measured rates of change.

### Cross-Sectional Data Underestimate Age-Related Brain Changes.

We found that median rates of age-related change were significantly lower for cross-sectional compared to longitudinal estimates across all four phenotypes: GMV [*t* = 19.23, *P_FDR_* < 0.05, false discovery rate (FDR) corrected across four phenotypes, *Cohen’s d* = 0.43]; CTh [*t* = 31.80, *P_FDR_* < 0.05, *Cohen’s d* = 0.71], SA [*t* = 40.20, *P_FDR_* < 0.05, *Cohen’s d* = 0.90], and FA [*t* = 19.89, *P_FDR_* < 0.05, *Cohen’s d* = 0.45]. Brain aging inferred from cross-sectional data (i.e., pseudo-longitudinal experimental designs) thus markedly underestimated age-related trends. Underestimation was most pronounced for CTh, with underestimation of up to 44% (at 60 y of age), followed by SA (37% underestimation at 72 y), FA (35% underestimation at 73 y), and GMV (26% underestimation at 73 y).

Underestimation was evident regardless of the timepoint (baseline or follow-up) used to construct cross-sectional normative models (*SI Appendix*, Fig. S1) and was more pronounced for centiles other than the median (see *SI Appendix*, Fig. S2 for 2%, 16%, 84%, and 98% centiles). Region-level analyses determined that underestimation was spatially diffuse, and overestimation was evident for a small fraction (<20%) of regions, including the SA of the right central sulcus and the middle-posterior segment of the right cingulate gyrus and sulcus, as well as CTh of the left calcarine sulcus (*SI Appendix*, Fig. S3). Furthermore, a supplementary analysis was conducted to determine whether cross-sectional underestimation occurs when estimating longitudinal rates of change, rather than directly modeling rates of change from longitudinal measurements. To this end, linear mixed effects (LME) models were fitted to infer rates of change using the longitudinal MRI measures while controlling for repeated measurements. LME-derived rates of change were compared to cross-sectional rates of change, as estimated from general linear models. This comparison recapitulated the underestimation by cross-sectional data (*SI Appendix*, Fig. S4), confirming that underestimation by cross-sectional is evident regardless of directly or indirectly (LME) deriving rates of change from longitudinal measurements. Lastly and perhaps most critically, underestimation of age-related change was replicated in the developmental (ABCD) cohort, revealing a maximal percent difference of 99% (*SI Appendix*). Our finding underscores the importance of calibrating brain reference charts with longitudinal data to improve accuracy in normative brain trajectories and to facilitate inference at an individual level.

### Cross-Sectional Normative Models Minimally Aid Individualized Prediction.

**Fig. 3.**
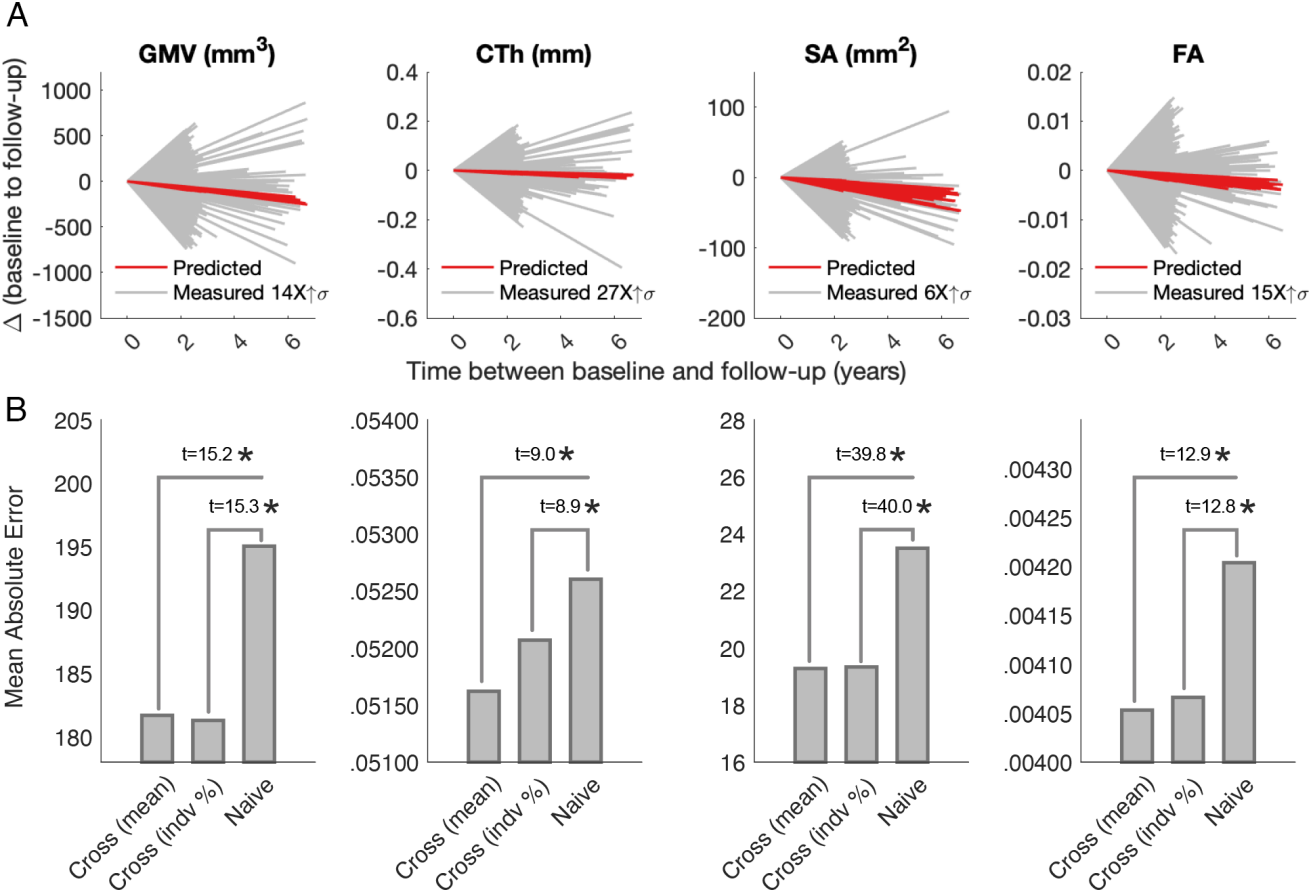
Predicting individualized trajectories from group-level cross-sectional trends. (*A*) Observed change from baseline to follow-up (*X* axis) and predicted rate of change, as estimated from cross-sectional (cross) baseline data (using the 50-th for all individuals). (*B*) Mean absolute error in predicting the rate of change with cross-sectional models based on the 50-th percentile (mean) and individualized percentiles at baseline (indv %) and naive models (i.e., follow-up phenotype values are equal to baseline phenotype values). Bars denote between-group comparisons and asterisks denote significance (*P_FDR_* < 0.01). Predictions derived from median centiles and individual-specific centiles yielded comparable accuracy for all phenotypes (no significant differences, *P* > 0.05). Abbreviations: gray matter volume (GMV); cortical thickness (CTh); surface area (SA); fractional anisotropy (FA); Cross (cross-sectional).

We next asked whether individual trajectories can be predicted using group-level rates of change inferred from cross-sectional normative models. To this end, we predicted follow-up brain phenotype measurements for unseen individuals using cross-sectionally inferred rates of change. An individual’s follow-up brain phenotype measurement was predicted such that ${\hat{y}}_{1}=y_{0}+\int_{age_{0}}^{age_{1}}\Delta_{q}\left(x\right)dx$ , where $y_{0}$ is the baseline measurement and $\Delta_{q}\left(x\right)$ denotes normative rate of change at age *x* years for the *q* th percentile. Fig. 3*A* shows the predicted (red lines) and empirically measured (gray lines) age-related trajectories for all individuals and brain phenotypes. Predictions were formed using: i) the median centile (*q* = 0.5) for all individuals (Fig. 3 *A* and *B*) or ii) the individual’s specific centile at baseline (*SI Appendix*, Fig. S5).

We found that the empirically measured variation of individual change was substantially (6 to 27 times) greater than our predictions (Fig. 3*A*). Approximately one-third of all individuals departed from the predicted downward direction of change in the aging cohort—the percentage of individuals with upward slopes was 34% for GMV, 42% for CTh, 21% for SA and 39% for FA. All model-based predictions outperformed naive prediction where follow-up measurements were predicted to be equal to baseline measurements (i.e., to test the null hypothesis that phenotypes remain fixed over time). Specifically, significant differences were observed in mean absolute errors (MAEs) between cross-sectional and naive predictions (Fig. 3*B*). Despite these significant effects, the percent reduction in prediction errors was 18% for SA, 7% for GMV, 4% for FA, and 2% for CTh, demonstrating that group models of MRI measures generated from cross-sectional data offer minimal improvement to predictions of individual change relative to naive models of no change. The performance of individualized prediction from cross-sectional rates of change did not significantly differ to an alternative null hypothesis constructed by randomizing subject age (±5 y) while preserving interscan interval (1,000 permutations). Specifically, there was no significant difference in MAEs between predictions based on age-correct cross-sectional rates of change and predictions based on age-shuffled cross-sectional rates of change (*P* > 0.05 across all phenotypes). Thus, we conclude that while normative models capture shared age variance, person-specific factors may outweigh the influence of age in determining individual rates of change.

### Nonage-Related Factors Contribute to Errors in Predicting Individualized Change.

**Fig. 4.**
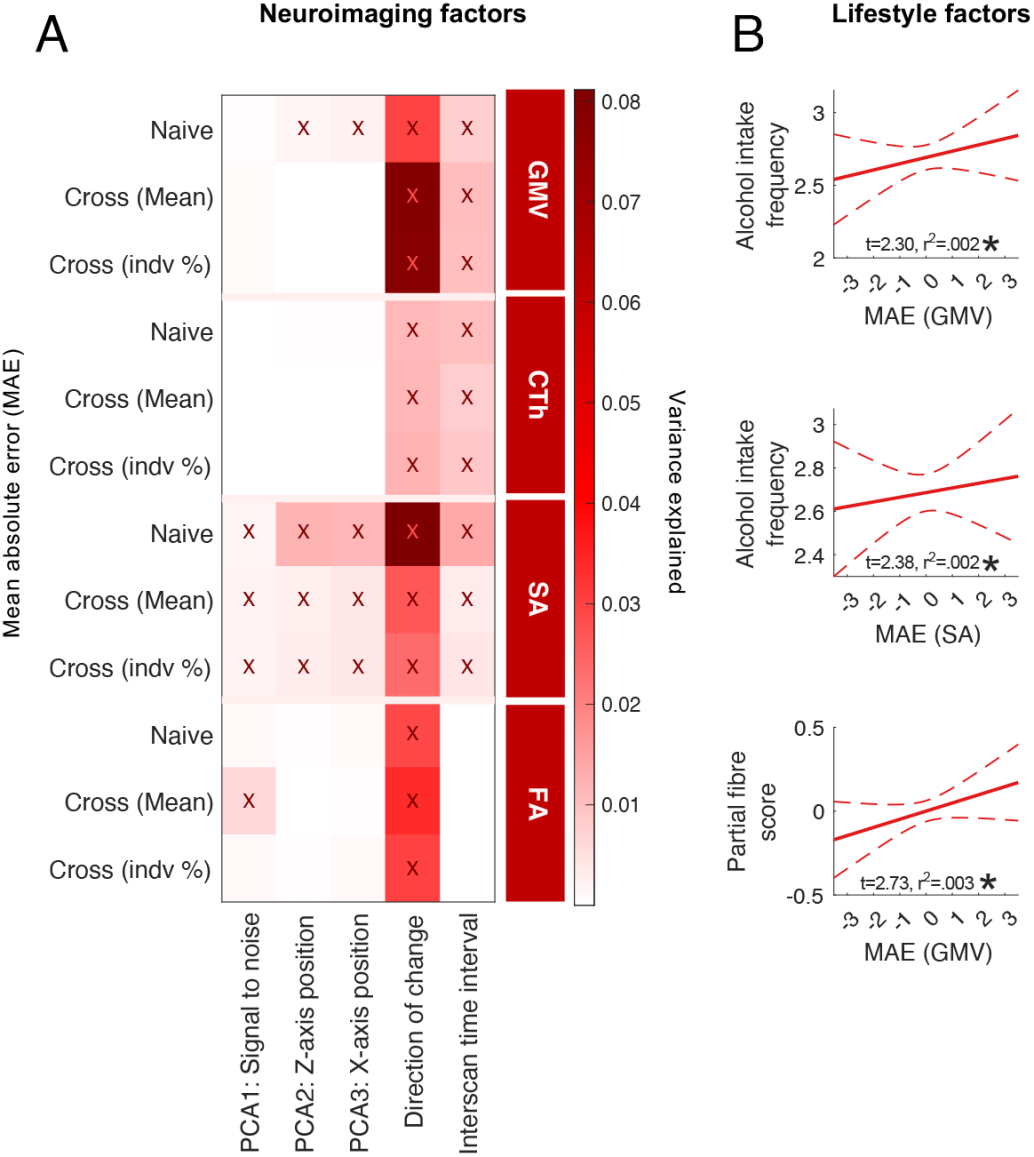
Neuroimaging and lifestyle associations with errors in predicting individualized rates of change. (*A*) MAE variance explained (r^2^) by neuroimaging factors, including PCA-summarized neuroimaging confounds measured at baseline (derivation of PCA components are shown in *SI Appendix,* Fig. S7), direction of change (increasing/decreasing), and interscan time interval (*B*) Regression plots present significant correlations between lifestyle measures (*Y* axis) and normalized MAE (*X* axis) from cross-sectional predications of change, based on the 50-th percentile (*X* axis). Asterisks denote significance (*P_FDR_* < 0.05). Abbreviations: Principal component analysis (PCA); mean absolute error (MAE); cross-sectional (cross); individual (indv).

Demographic, neuroimaging, developmental, and lifestyle factors are known sources of nonage-related variability (10–13). MAEs in predicting individualized change were significantly higher in males relative to females and across all models examined for GMV and SA but not for CTh and FA (*SI Appendix*, Fig. S6). In contrast, no difference in MAE was found between individuals of and not of European ancestry or between individuals with and without a medical/psychiatric/neurologic diagnosis (*SI Appendix*, Fig. S6). Fig. 4*A* shows associations between MAE with neuroimaging-related factors, including MRI-related confounds [three principal component analysis (PCA)-derived components as specified in *SI Appendix*, Fig. S7 and Table S2], the direction of observed change from baseline to follow-up, and interscan time interval. Poorer prediction accuracy was significantly associated with neuroimaging confounds (Fig. 4*A*), upward (rather than downward) slopes in the direction of change, and longer interscan intervals for T1 measures (refer to *SI Appendix*, Fig. S8 for correlation plots). The percentage of error variance explained by any one neuroimaging variable was small (<1%), implying additional determinants of individual variability in age-related change estimates. We thus examined a range of potential lifestyle-related sources underlying nonage-related variability, including features of alcohol consumption, physical activity, sleep, and tobacco smoking. As shown in Fig. 4*B*, prediction errors from individualized estimates of age-related change in GMV and SA were significantly and positively correlated to alcohol intake frequency and partial fiber scores, indicating that greater frequency of alcohol consumption and lower dietary intake of fiber aid the prediction of individualized age-related GMV trajectories. Lifestyle correlations were consistent between cross-sectional models based on the 50-th percentile (mean) and individualized percentiles at baseline (indv %).

## Discussion

In the age of precision medicine, the goal of neuroimaging in psychiatry and neurology extends beyond static and group-level inference to include dynamic predictions of individualized change (16). We examined whether current MRI data and methodological approaches can realize such ambitions. Utilizing state-of-the-art developmental and aging datasets, we show that cross-sectional aging models underestimate longitudinal measurements of change in standard MRI brain measures. This divergence between intra- and inter-person trajectory estimates varies with age and specific brain measures. We also show that group-level trends offer significant, albeit minor, improvements to naive predictions of individualized change based only on an individual’s brain measurement at baseline.

Fundamental knowledge of macroscale brain development and aging relies on accurate age trends. Our findings cohere with preliminary evidence that cross-sectional age trends underestimate longitudinal changes across 5 years in regional volumetric measures (4). Here, we extend this conclusion to multiple brain modalities and age-diverse populations, using timely normative models and large-scale biobank resources. An implication of these findings is that imprecise estimates provide shaky foundations for prediction (e.g., critical periods of growth/decline, disease outcomes, neurodegenerative decline, etc) and downstream hypotheses regarding the biology and impacts of aging (24, 25). For example, cross-sectionally inferred trajectories of SA (Fig. 2*B*) are relatively stable compared to longitudinal estimates that highlight increasing rates of change with advancing age. Based on the cross-sectional normative model, researchers may wrongly conclude that SA is preserved in the aging process or that other variables, such as those related to cognition, decline independently of SA. In terms of applying normative models to monitor individualized trajectories, an individual may be wrongly referred for follow-up clinical investigation because their brain has been assessed as exhibiting rapid decline relative to established norms.

The critical issue with respect to the interpretation of age trends is whether time-related effects on the brain varied as a function of age. Time and age effects are inextricably intertwined, particularly in age-diverse cohorts where brains may harbor signatures of time due to, for example, major historical events or medical treatments available at a given time. We found less pronounced age-related change in estimates derived from cross-sectional data when compared to longitudinal data, across all brain measures examined. This effect was spatially diffuse at a regional level, such that only a small number of regions did not significantly differ from or overestimated longitudinal age trends. As such, time-related effects associated with the period of measurement (or measurement errors) generally outweighed variability ascribed to age. Critically, underestimation of age-related change by cross-sectional normative models in both an age-diverse (UKB age range = 47 to 80 y) and age-constrained (ABCD age range = 9 to 11 y) cohort, indicating that factors beyond cohort/generational effects likely contribute to the time-related impacts observed in longitudinal rates of age-related change. Age-related effects as observed naturally across time are nuanced by individual aspects related to the brain structure.

Our findings indicate that normative models of MRI measures generated from cross-sectional data offer only minimal improvement to predictions of individual change relative to naive models of no change. This finding may be unsurprising given that many individuals displayed trajectories that opposed group-level trends, consistent with previous studies. For example, in contrast to an expected age-related decline in CTh with advancing age, longitudinal studies have reported increases in CTh over time, which may relate to exercise (26), peripheral telomerase activity (27), and at a cellular level, to enhanced gliogenesis or proliferative capacity of critical support cells in the brain (27). Thus, while normative models capture shared age variance that accords with the cellular hallmarks of brain maturation and decline, person-specific factors outweigh the influence of age in determining individual rates of change. These findings should not be interpreted as diminishing the importance of cross-sectional studies. Cross-sectional data are critical for examining group differences, brain regional variability, and brain associations with individual factors. Instead, our findings suggest that longitudinal data offer complementary characterization of intraindividual variation to reveal nonage-related determinants of change.

Dynamic changes in MRI measures relate to numerous person-specific factors that in turn, impact prediction accuracy. We found that prediction errors in estimating individual rates of change reflected person-specific factors including noise related to MRI confounds and direction of observed change and interscan interval, as well as key lifestyle determinants. Interestingly, prediction accuracies in individual age trajectory estimates of GMV and SA were aided by factors linked to poor health, including higher overall alcohol intake and lower dietary fiber intake. For example, intake of dietary fiber—nondigestible forms of carbohydrate that usually originate from plant-based foods—has been inversely associated with the risk of dementia (28), cognitive impairments (29), and total brain volume (30). Therefore, we speculate that factors accelerating typical age-related brain trends (i.e., exaggerated brain atrophy with advancing age) improves prediction accuracy by way of increasing the likelihood that brain changes track along the same direction as normative brain age trends. In contrast, predictions were less accurate in individuals that depart from typical brain age trends. These findings call into question the ability of established reference norms—particularly in adolescence and adulthood—to accurately predict individualized patterns of change. Uncovering person-specific factors that improve prediction accuracies will likely aid the clinical utility of normative brain charts in psychiatry and neurology settings.

We note the following limitations. Brain trajectory estimates may be subject to selection bias, as the likelihood of participating in follow-up assessments is nonrandom. It is possible, for example, that individuals participated in follow-up brain imaging due to a family history or personal/subjective concerns about cognitive decline and neurodegeneration, manifesting in greater brain changes in this group relative to the wider population imaged at baseline (31). While we cannot determine these effects, our results that indicate underestimation by cross-sectional-indexed rates of change were replicated in an independent developmental cohort, where selective loss-to-follow-up was acceptably low (32). Another important consideration is that our longitudinal models still encompass a cross-sectional component, such that rates of change modeled as a function of age were not drawn from repeated MRI measurements evenly sampled across 30 y in the same individuals. Therefore, our longitudinal models remain subject to the pitfalls inherent to cross-sectional designs (e.g., generational effects), albeit to a lesser extent. Pure longitudinal designs would undoubtedly derive more accurate estimates of age-related change and potentially yield stronger correlations with various lifestyle, genetic, or neurobiological variables. Nonetheless, our findings suggest that even in the context of longitudinal models biased by cross-sectional influence, cross-sectional underestimation of age-related change is pronounced, particularly for ages marked by dynamic change, in both an aging and developmental cohort. This is due to person-specific factors that outweigh the common influence of biological age-related processes on MRI measures.

Major gaps remain in our knowledge around human brain aging trajectories, particularly as inferred from cross-sectional data. We outline three specific recommendations that follow from our findings, and that would benefit from further research:

1. Much of what is known about trajectories of brain aging derives from cross-sectional investigations. Our results here suggest that cross-sectional models and brain charts can underestimate the extent of age-related brain changes. Caution is thus recommended when interpreting cross-sectional estimates, and calibration/correction factors may be required. Prospective studies will be needed to enable calibration of cross-sectional models and to improve their accuracy for individual and prospective inference.
2. Our work challenges the assumption that neuroimaging confounds constitute a substantial share of variance in errors from predicting individualized trajectories of brain change. Furthermore, a range of demographic, developmental, and lifestyle factors, respectively contributed less than 2% of variance. Further exploration into types and sources of error in predicting longitudinal change from cross-sectional rates of change will enhance the practical utility of future normative models.
3. A considerable portion of subjects were found to exhibit trajectories of brain change that defy average age trends The potential influence of measurement error (resulting from sources unexamined here) and/or possible biological bases of these changes warrants further exploration to expand knowledge around lifelong neuroplasticity, as well as the determinants and benefits/detriments of contrary brain age trajectories.

Normative models provide new opportunities to harness neuroimaging in clinical settings, for example, by providing benchmarks against which individuals can be monitored for neurodegenerative disorders in vivo. However, individual-centric approaches present new challenges in terms of accuracy and reliability. Our findings demonstrate that normative models established on cross-sectional data underestimate group-level age trends and that group-level normative models are limited in terms of individualized inference. Before we can realize the ambitions of normative models, it is first prudent to address these barriers impeding their utility.

## Materials and Methods

*SI Appendix* details sample characteristics, the derivation and treatment of study variables, age-related brain age models, and statistical testing. All data were drawn from the UKB and the ABCD. UKB approval was obtained from ethics committees as detailed in http://www.ukbiobank.ac.uk/ethics/. ABCD approval was obtained from a centralized institutional review board (IRB) within the University of California, San Diego and from local IRBs obtained from each study site. Written informed consent was obtained from each participant (and from parents in the ABCD).

### Modeling Age-Related Change.

Normative centile curves were fit to whole-brain MRI estimates of GMV, CTh, SA, and FA using GAMLSS implemented in R v2021.09.1 (Build 372) (33, 34). The GAMLSS framework is a semiparametric normative modeling framework that accounts for heteroscedasticity, non-Gaussian distributions, and nonlinear trajectories. For each phenotype, the GAMLSS model (*SI Appendix*) using a Box-Cox t distribution—a shifted and truncated version of the t distribution (35)—was fitted separately to i) cross-sectional data and ii) longitudinally estimated rates of change (see *SI Appendix*, Fig. S9 for QQplots visualizing GAMLSS model fits). The cross-sectional rate of change was inferred by numerically differentiating the *k*-th percentile as a function of age for the GAMLSS curve. Expressed as a percentage, the cross-sectional rate of change at $age=(age_{n}+age_{n+1})/2$ was thus given by,

$$\Delta_{cross}^{k}\left(age\right)=\frac{{\overset{-}{y}}^{k}\left(age_{n+1}\right)-{\overset{-}{y}}^{k}\left(age_{n}\right)}{age_{n+1}-age_{n}}\times \frac{100\%}{{\overset{-}{y}}^{k}\left(age_{n}\right)},$$

where ${\overset{-}{y}}^{k}\left(age_{n}\right)$ is the *k*-th percentile at $age_{n}$ estimated from the GAMLSS fitted to the baseline data. The age range was uniformly sampled at a resolution of $age_{n+1}-age_{n}\approx$ 2 wk (see *SI Appendix*, Fig. S10 for results across alternative sampling resolutions). Given that differentiation is a sharpening operation that can amplify noise, $\Delta_{cross}^{k}$ was smoothed as a function of age using a moving average filter with a span of approximately ±2.5 y.

### Predicting Individual Phenotype Measurements at Follow-Up Using Rate of Change Estimates.

The group-level cross-sectional ( $\Delta_{cross}^{k}$ ) estimates of rate of change were used to predict *individual* phenotype measurements at follow-up. For the *i*-th individual with baseline and follow-up phenotype measurements at $age_{n}$ and $age_{n+1}$ , the follow-up phenotype measurement was predicted from the baseline measurement according to the integral,

$${\hat{y}}_{i}\left(age_{n+1}\right)=y_{i}\left(age_{n}\right)+\underset{age_{n}}{\int^{age_{n+1}}}\Delta_{cross}^{k}\left(x\right)dx.$$

The integral was evaluated using the trapezoidal rule with a spacing of approximately 2 wk between successive estimates of rate of change. Predictions were computed using i) the 50-th percentile ( k=50 ) for all individuals and ii) individualized percentiles determined by the individual’s percentile at baseline. In the above integral, estimated rates of change ( $\Delta_{cross}^{k}$ ) were expressed in absolute terms, not as percentages. Prediction accuracy was measured using the MAE, given by,

$$MAE=\frac{1}{N}\sum_{i=1}^{N}AE_{i},\;AE_{i}=\left|{\hat{y}}_{i}\left(age_{n+1}\right)-y_{i}\left(age_{n+1}\right)\right|.$$

MAE was computed for cross-sectional predictions as well as a *naive prediction* where ${\hat{y}}_{i}\left(age_{n+1}\right)=y_{i}\left(age_{n}\right)$ for all individuals i=1,⋯,N.

## Supplementary Material

Appendix 01 (PDF)

## Data Availability

MRI data are available from the UKB Access Management System (https://www.fmrib.ox.ac.uk/ukbiobank) (36) and the ABCD data repository (https://data-archive.nimh.nih.gov/abcd) (37). Normative modeling was performed using GAMLSS frameworks, which is publicly available code in R v2021.09.1 (Build 372). All codes are available on GitHub: https://github.com/mdibiase1/Predict_Rate_of_Change.git.
