# Supplementary material for: Mapping human brain charts cross-sectionally and longitudinally
Source: Proc Natl Acad Sci U S A. 2023 May 8;120(20):e2216798120. doi: 10.1073/pnas.2216798120 (PMC10193972; doi:10.1073/pnas.2216798120)
Supplement: Supplementary file 1 — Appendix 01 (PDF) [file pnas.2216798120.sapp.pdf]

Supporting Information for
Mapping human brain charts cross-sectionally and longitudinally

Maria A. Di Biase, Ye Ella Tian, Richard A. I. Bethlehem, Jakob Seidlitz, Aaron. F. Alexander-Bloch, Thomas Yeo & Andrew Zalesky

Maria A. Di Biase
Email: dibiasem@unimelb.edu.au

This PDF file includes:

Supporting text
Figures S1 to S14
Tables S1 to S4
SI References

Supporting Information Text

Extended Methods

Samples.

UK Biobank (UKB). UKB is an open-access resource comprising data from 502 486 individuals between age 40 and 69 years at baseline assessment (1). Individuals were recruited between 2006 and 2010 from 22 centers across the United Kingdom to capture heterogeneous socioeconomic demographics and a mixture of urban and rural residents. Participant and data collection details are provided elsewhere (1). This study utilized data from a subset of individuals with available baseline and follow-up MRI data (from the Imaging visit [2014+] and first repeat (follow-up) imaging visit [2019+]). Table S1 presents sample characteristics for the sample utilized in this study. Approval for UKB data collection was obtained from appropriate ethics committees (http://www.ukbiobank.ac.uk/ethics/). Written informed consent was obtained from each participant. All ethical regulations were complied with during data collection and analysis.

Adolescent Brain Cognitive Development (ABCD). The ABCD study is an open access resource comprising data from 11,877 individuals between age 9 and 10 years at the baseline assessment (4.0 Data Release [11877]). Individuals were recruited from 21 data sites across the U.S (2). Participant and data collection details are provided elsewhere (https://abcdstudy.org/scientists/protocols/). This study utilized data from a subset of participants with available baseline and follow-up MRI data. Table S1 presents sample characteristics for the sample utilized in this study. Approval for ABCD data collection was obtained from a centralized institutional review board (IRB) within the University of California, San Diego and from local IRBs obtained from each study site. Written informed consent was obtained from each parent and each child. All ethical regulations were complied with during data collection and analysis.

Image-derived phenotypes

UKB. Magnetic resonance imaging (MRI) data were acquired on a Siemens Skyra 3T running VD13A SP4 with a standard Siemens 32-channel RF receive head coil (3). T1-weighted MPRAGE, T2-weighted FLAIR and diffusion-weighted MRI (dMRI) volumes were acquired with parameters described elsewhere (3). This study utilized MRI phenotypes generated by an image-processing pipeline developed and run on behalf of UK Biobank (4), which primarily utilized Freesurfer v6.0 (5), and FMRIB Software Library (FSL) v5.0.10 (6, 7), as detailed elsewhere (4, 8). To yield whole-brain phenotypes, cortical gray matter volume (GMV; Data-Fields 27205-27235 & 27300-27328), cortical thickness (CTh; Data-Fields 27174-27204 & 27267-27297), and surface area (SA; Data-Fields 27143-27173 & 27236- 27266) were averaged across 31 regions comprising the Desikan-Killiany–Tourville (DKT) atlas (9). Regional weighted mean fractional anisotropy (FA) was averaged across 27 major white matter tracts defined by AutoPtx (10) (Data-Fields 25488-25514).

ABCD. MRI data were collected on three 3 Tesla (T) scanner platforms: Siemens Prisma, General Electric 750 and Phillips. T1-weighted MPRAGE, T2-weighted, and dMRI volumes were acquired as detailed elsewhere (11). This study utilized measures of GMV, CTh and SA, as well as white matter FA, which were generated by the ABCD team (12) using Freesurfer v5.3 (5), Analysis of Functional NeuroImages (AFNI)(13), and FSL (6, 7).

Treatment of MRI phenotypes. This study utilized whole-brain MRI-derived estimates (GMV, CTh, SA and FA) acquired at baseline and follow-up as well as the rate of change in each phenotype (follow-up minus baseline divided by interscan interval). Thus, negative rates of change indicate phenotypic decline across time. Interscan intervals are shown in Table S1 for both samples. For each phenotype, we excluded individuals with data that were outliers (defined as values more than three scaled median absolute deviations away from the median) on whole brain estimates or on rate of change. Table S3 summarizes sample filtering for each dataset and phenotype, respectively.

Lifestyle Variables

Alcohol. In a self-administered touchscreen questionnaire conducted at baseline, participants were asked to classify their current drinking status (never, previous, current, prefer not to say). Current drinkers were asked additional questions regarding their alcohol intake of a range of alcoholic drink types (red wine, white wine, champagne/white wine, spirits, beer/cider, and fortified wine). Using this information, three measures were derived: (1) *Weekly units consumed*(14) (summed across all drink types [Data-Fields 1568, 1578, 1588, 1598 & 1608]), (2) *Alcohol intake group*(15, 16) (units/week were used to classify participants into five categories: 1=nondrinker; 2=previous drinker; 3=within guidelines [<14 UK alcohol units/week for women and <21 units/week for males]; 4=hazardous [14–34 units/week in women and 21–48 units/week in men]; and 5=harmful [>35 units/week in women and >49 units/week in men]), and (3) *Alcohol intake frequency* (1=daily or almost daily; 2=three or four times a week; 3=once or twice a week; 4=one to three times a month; 5=special occasions only; 6=Never [Data-Field 1558]).

Diet. In the *UKB Food Frequency Questionnaire* conducted at baseline, participants were asked to report their intake of a range of foods (cooked vegetables, salad/raw vegetables, fresh fruit, dried fruit, oily fish, non-oily fish, processed meat, poultry, beef, lamb/mutton, pork, bread, cereal) and drinks (tea, coffee, and water). Using this information, six dietary measures were derived: (1) *Total vegetable intake* (summed cooked and salad/raw vegetable intake [Data-Fields 1289 & 1299]), (2) *Total fruit intake* (summed fresh and dried fruit intake [Data-Fields 1309 & 1319]), (3) *Total unprocessed red meat intake* (summed beef, lamb/mutton, and pork intake [Data-Fields 1369, 1379 & 1389]), (4) *Total fish intake* (summed oily and non-oily fish intake [Data-Fields 1329 & 1339]), (5) *Healthy diet score* (1=healthy; 0=unhealthy), and (6) *Partial fibre score*. *Healthy diet scores* were calculated in accord with previous research (17) based on dietary recommendations for cardiometabolic health (18). A score of 1 was based on consumption of at least four of the following 7 food groups: (1) Fruits (≥ 3 servings/day), (2) Vegetables (≥ 3 servings/day), (3) Fish (≥2 servings/week), (4) Processed meats (≤ 1 serving/week), (5) Unprocessed red meats (≤ 1.5 servings/week), (6) Whole grains (≥ 3servings/day), and (7) Refined grains (≤1.5servings/day). *Partial fibre scores* were constructed as previously detailed (19) using questions on fruit, vegetables, bread, and cereal (type and intake). Food groups were assigned portion sizes, based on (20) and non-starch polysaccharide content based on (21). For each food item, fibre content was multiplied by consumption frequency (‘less than one’ was coded as 0.5). Fibre intake was then summed across food groups to estimate daily *partial fibre intake* scores. The estimated fibre content of each food item and Data-Fields used to generate the partial fibre score are detailed elsewhere (19).

Physical activity. In a modified version of the *International Physical Activity Questionnaire* (IPAQ) conducted at baseline, participants were asked to report on frequency (# days), duration (minutes), and type of physical activity (walking [Data-Fields 864 & 874], moderate [Data-Fields 884 & 894], and vigorous [Data-Fields 904 & 914]) performed over the last 7 days. This information was used to derive two physical activity measures: (1) Metabolic Equivalent Task (MET) minutes per week (continuous score constructed using IPAQ guidelines (22)), and (2) MET group. In line with IPAQ guidelines (22), all physical activity time variables exceeding 180 minutes/3hours were truncated to be equal to 180 minutes. Participants were dived into three mutually exclusive groups based on standard IPAQ scoring criteria (22): 1=low physical activity (<600 MET-min/week), 2=moderate physical activity (vigorous-intensity activity on ≥3 days of ≥20 minutes/day OR moderate-intensity activity and/or walking on ≥5 days of ≥30 minutes/day OR moderate-intensity activity and/or walking on ≥5 days of ≥30 minutes/day OR any combination of activities on ≥5 days achieving ≥600 MET-minutes/week), and 3=high physical activity (vigorous-intensity activity on ≥3 days achieving ≥1500 MET-minutes/week OR more days of any combination of activities achieving ≥3000 MET-minutes/week).

Sleep. In the self-administered touchscreen questionnaire conducted at baseline, participants were asked seven sleep-related questions, which were used to derive six measures used in this study: (1) Sleep duration (continuous score [derived from Data-Field 1160]), (2) Sleep duration group (1=short [<7hours]; 2=normal [7-8hours]; 3=long [>8hours; derived from Data-Field 1160]), (3) Chronotype (1=definitely morning; 2=morning>evening; 3=evening>morning; 4=definitely evening [Data-Field 1180]), (4) Sleepiness/Insomnia (1=usually; 0=never or

sometimes [derived from Data-Field 1200]), (5) Snoring (1=yes; 0=no [Data-Field 1210]), and (6) Daytime dozing/Narcolepsy (1=all of the time or often; 0=never, rarely or sometimes [derived from Data-Field 1120]).

Smoking. In the self-administered touchscreen questionnaire conducted at baseline, participants were asked whether they ever smoked tobacco (Data-field 20160). Participants endorsing a 'yes' response were asked additional tobacco smoking-related questions, which were used to derive four measures based on previous research (23): (1) current smoking status (1=currently smokes on most or all days; 0=non-smoker or smokes occasionally [derived from Data-Field 1239]), (2) ever smoked (1=current or former smoking on most or all days, 0=never smoking on most or all days, [derived from Data-Field 1239]), (3) cigarettes per day (present if they are currently smoking [Data-Field 3456] or past if they are no longer smoking [Data-Field 2887]), and (4) smoking duration (age at the time of data collection [Data-Field 21003] minus the year they started smoking [Data-Field 3436] if they are currently smoking, and the year they stopped smoking [Data-Field 2897] minus the year they started smoking [Data-Field 3436] if they are no longer smoking). Cigarettes per day and smoking duration were only analysed among those with >0 cigarettes per day and >0 years of smoking, respectively.

Modeling age-related change. Normative centile curves were fit to whole-brain MRI estimates of GMV, CTh, SA and FA using GAMLSS implemented in R v2021.09.1 (Build 372) (24, 25). The GAMLSS framework is a semi-parametric normative modelling framework that accounts for heteroscedasticity, non-Gaussian distributions, and nonlinear aging trajectories. Individual variation in an IDP denoted with y was modeled such that,

$$y = BCT(\mu, \sigma, \nu, \tau)$$

$$\mu = f_\mu^1(\text{age} \times \text{female}) + f_\mu^2\ (\text{age} \times \text{male}) + \beta_\mu z_{\text{site}}$$

$$\sigma = f_\sigma(\text{age}) + \beta_\sigma z_{\text{site}}\ , \qquad \nu = \beta_\nu, \qquad \tau = \beta_\tau\ .$$

where BCT denotes the Box-Cox t distribution—a shifted and truncated version of the t distribution (26). The distribution was parameterized with four parameters: median/location (μ), centile-based coefficient of variation (σ), skewness (ν) and kutosis (τ). Several alternative four parameter candidate distributions were investigated and the BCT was found to yield the best fit, evaluated using the BIC (see *SI Appendix* for assessment of model fit). Modeling the effects of age and sex on the skewness and kurtosis led to models of greater complexity with marginal improvements in model fit. Hence, constants were fitted for skewness and kurtosis. The location parameter also modeled the effect of neuroimaging covariates, including positions of center-of-gravity and table/coil, intensity scaling, and signal-to-noise and contrast-to-noise ratios (*SI Appendix,* Table S2 presents a complete list of neuroimaging confounds). For IDPs with more than three neuroimaging confounds, principal component analysis (PCA) was used to reduce dimensionality into three components that captured the most variance, as described in (27).

To assess model fit, the quantiles of the fitted distribution for each individual measurement were mapped to z-scores of a standard normal distribution and Q-Q plots were used to visually assess that the z-score normality. The Kolmogorov–Smirnov (KS) test was used to confirm the normality of z-scores for all phenotypes (refer to Figure S9 for QQ plots and Table S4 KS results). Additionally, goodness of model fit was determined by minimizing the Akaike Information Criterion (AIC) index.

Statistical testing. Permutation-based two-sample t-tests (1000 permutations) were used to assess the null hypothesis of equality in the mean absolute error (MAE) between cross-sectional and naïve predictions of follow-up phenotype measurements. On each permutation, rates of change were randomly assigned to cross-sectional and longitudinal groups and a t-test performed to construct an empirical null distribution. Permutation-based general linear models (GLMs; 1000 permutations) were formulated to assess relationships between variation in cross-sectional prediction errors (i.e., MAE) with demographic factors (sex, ethnicity, and diagnostic status),

neuroimaging factors (neuroimaging confounds, direction of change in brain phenotypes from baseline to follow-up [1=increase; 0=decrease], and interscan time interval) and lifestyle factors (alcohol, diet, sleep, physical activity, and smoking). The null distribution was constructed by randomizing MAEs across subjects to identify significant associations, with the false discovery rate (FDR) used to enforce control over multiple comparisons. Associations with neuroimaging factors controlled for age and sex. Error-lifestyle associations controlled for age, sex, genetic grouping (Data Field 22006), social economic status (Townsend Deprivation Index), site (2 sites in total), and three PCA-derived components characterizing neuroimaging confounds. Associations were examined using data from individuals without missing data for each variable respectively.

Extended Results

Results in the developmental cohort (ABCD dataset).

In the developmental cohort, all four brain phenotypes displayed a linear increase with age (Figure S11). Consistent with the aging dataset, cross-sectional data underestimated annual rates of change mapped from longitudinal data across GMV (t=5.29, *pFDR*<0.05, d=0.12); CTh (t=15.03, *pFDR*<0.05, d=0.33), and FA (t=24.80, *pFDR*<0.05, d=0.56). The discrepancy displayed trend-level significance in SA (t=1.76, *pFDR*=0.07, d=0.04). Disagreement between cross-sectional and longitudinal data was at 14 years for GMV (73%), 9 years for CTh (17%), 11 years for SA (99%), and 14 years for FA (73%). Consistent with the aging cohort, a considerable portion individuals departed from the group average predicted direction of change for each brain measure respectively (14% for GMV, 13% for CTh, 43% for SA and 22% for FA). In addition, prediction estimates for age-related change were marginally albeit significantly improved by cross-sectional population models relative to naïve models of no change (Figure S12). In contrast, the naïve model marginally outperformed cross-sectional predictions for rates of change in SA. This reflects substantial variability in observed SA change, marked equally by upward and downward slopes. Consequently, each subject's empirical baseline data (the basis of naïve prediction), was more accurate than cross-sectional predictions based on modelled centiles. MAEs in all brain phenotypes were associated with person-specific demographic factors (Figure S13) neuroimaging confounds (Table S2) and longer interscan intervals (Figure S14).

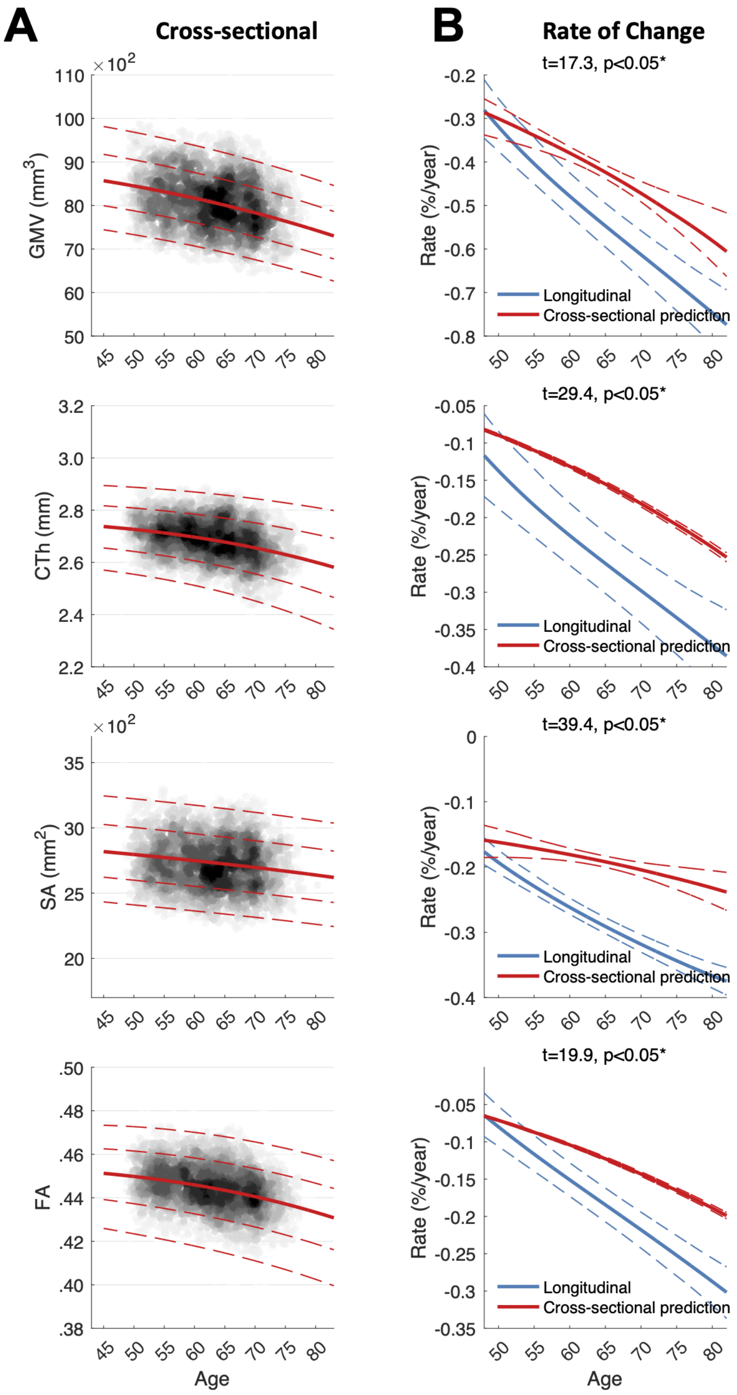

Fig. S1. Discrepancies between cross-sectional and longitudinal estimates of rates of change are robust against timepoint used to yield cross-sectional models (UKB dataset). Normative centile reference ranges for **(A)** cross-sectionally measured whole-brain GMV, CTh, SA and FA and **(B)** rates of change for each phenotype. Red curves in **(A)** and **(B)** were generated by modeling a randomly selected timepoint for each subject (thereby breaking the longitudinal relationship). (B) shows 25% and 75% confidence intervals (dashed lines), generated with bootstrapping (500 samples). **Abbreviations:** gray matter volume (GMV); cortical thickness (CTh); surface area (SA); fractional anisotropy (FA).

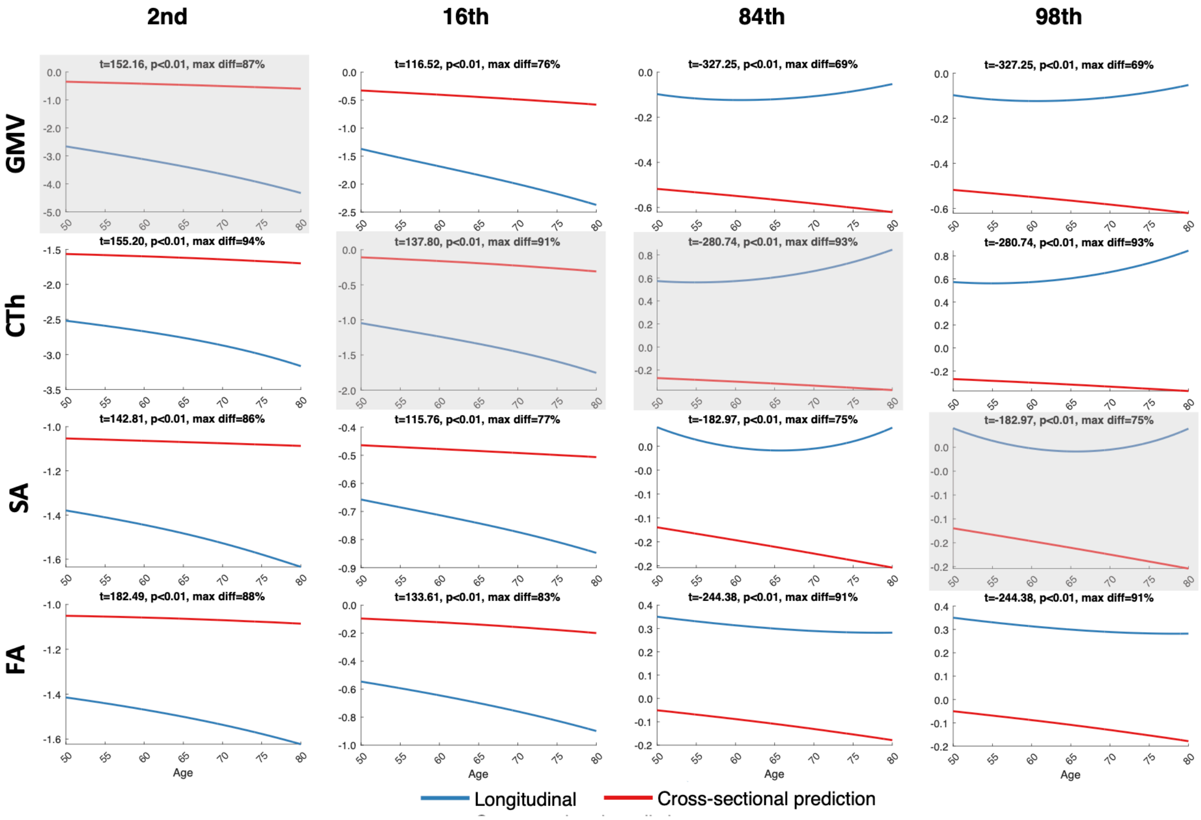

Fig. S2. Discrepancies between cross-sectional and longitudinal estimates of rates of change shown for other centiles (UKB dataset). Annual rate of change as estimated from longitudinal data (blue) and cross-sectional baseline data (red) as a function of age (x-axis). Gray boxes highlight centiles with the highest discrepancy for each MRI phenotype, based on maximum percent difference. **Abbreviations:** gray matter volume (GMV); cortical thickness (CTh); surface area (SA); fractional anisotropy (FA).

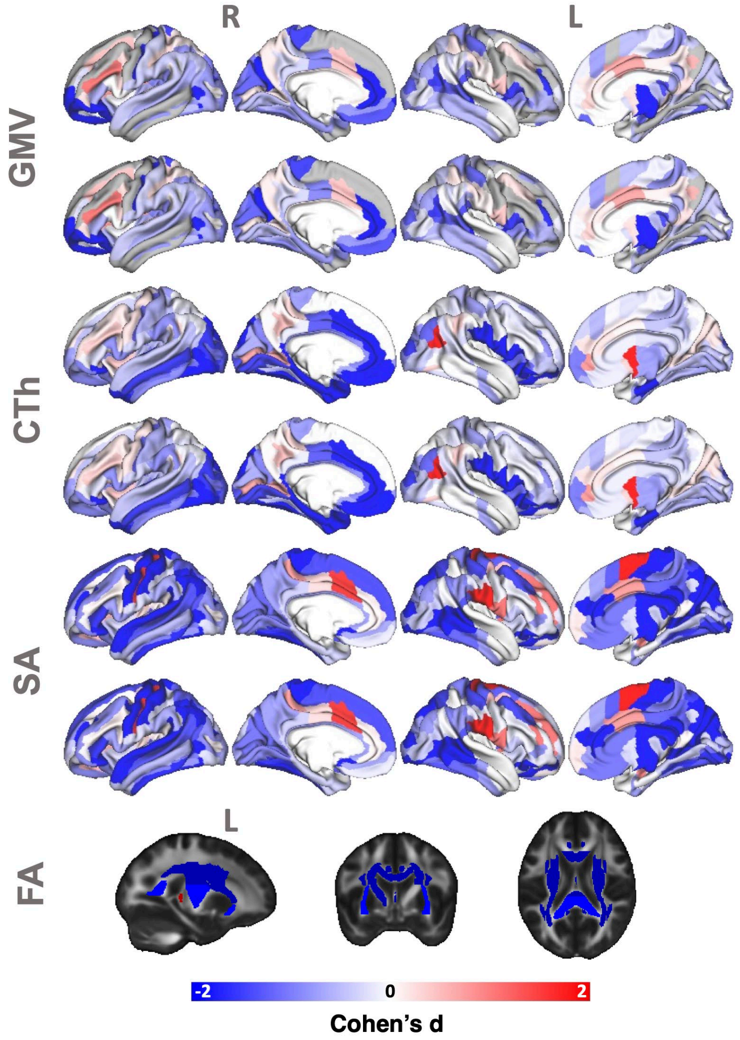

Fig. S3. Region-level discrepancies between cross-sectional and longitudinal estimates of rates of change (UKB dataset). Surface renderings display effect size (Cohen's d) estimates for regions displaying significant differences between cross-sectionally- and longitudinally-derived median rates of age-related change (after FDR correction for multiple comparisons across brain regions). Note that negative Cohen's d values (blue) reflect underestimation by cross-sectional estimates. Region-level brain phenotypes were previously generated by the UKB: gray matter volume (GMV; Data-Fields 27477-27550 & 27699- 27772), cortical thickness (CTh; Data-Fields 27403-27204 & 27625- 27698), and surface area (SA; Data-Fields 27329-27402 & 27551- 27624) derived from the Destrieux atlas (28) were used to align with region-level ABCD data (9). Regional fractional anisotropy (FA) values were derived from the JHU ICBM DTI-based white matter atlas (29). **Abbreviations:** gray matter volume (GMV); cortical thickness (CTh); surface area (SA); fractional anisotropy (FA).

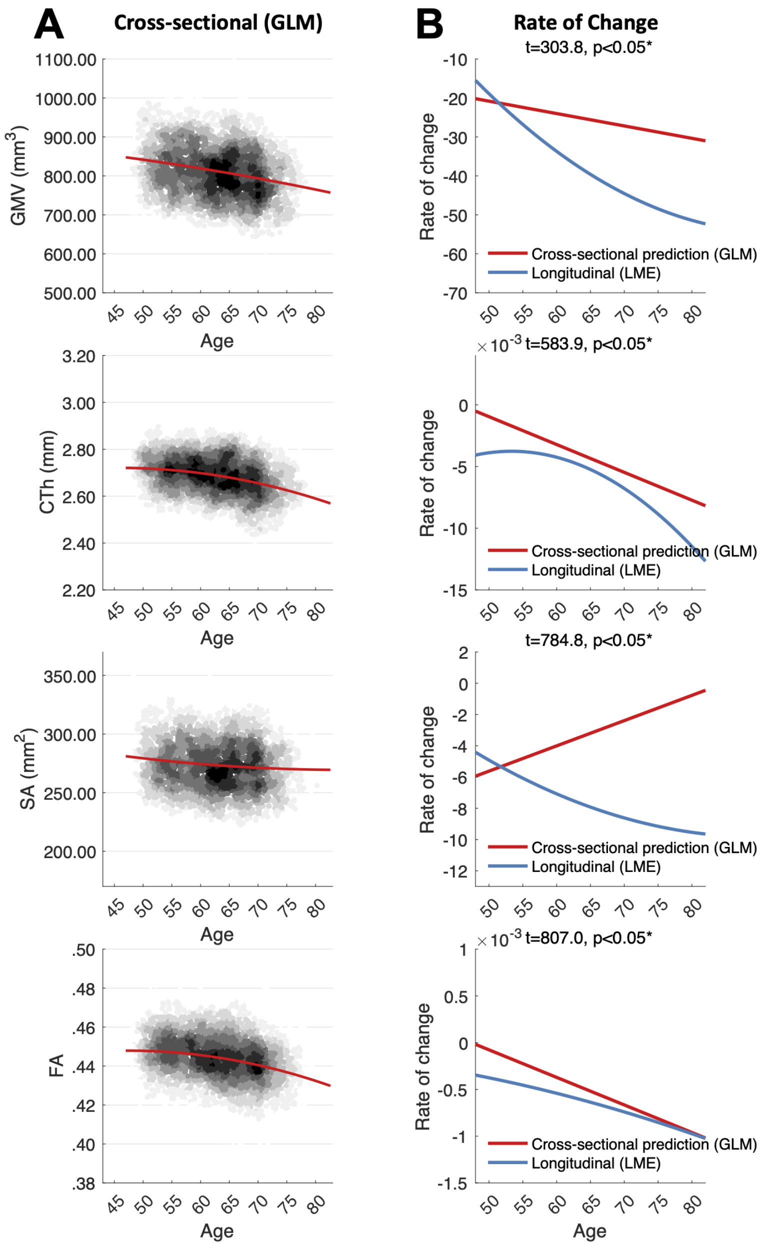

Fig. S4. Discrepancies between cross-sectional and longitudinal estimates of rates of change using general linear and linear mixed effects models (UKB dataset). General linear model estimates of **(A)** cross-sectionally measured whole-brain GMV, CTh, SA and FA and **(B)** rates of change for each phenotype. Rates of change were estimated: i) directly from longitudinally measured phenotypes using a linear mixed effects model (LME; blue); and ii) indirectly from cross-sectional data using general linear models (GLM; red). Rates of age-related change were significantly lower for cross-sectional estimates relative to longitudinal estimates across all four phenotypes. Asterisks denote significant differences (false discovery rate and 1000 permutations). Age and age-squared terms were modeled in both cross-sectional (GLM) and longitudinal (LME) models. **Abbreviations:** gray matter volume (GMV); cortical thickness (CTh); surface area (SA); fractional anisotropy (FA).

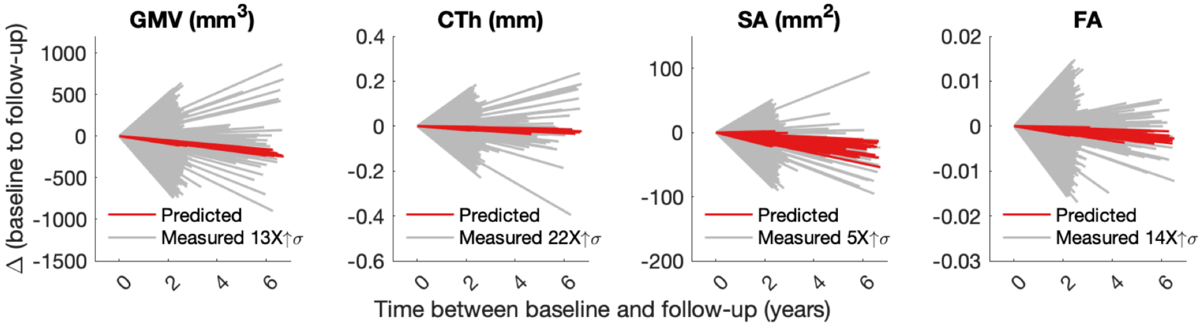

Fig. S5. Predicting individualized trajectories from group-level cross-sectional models based on individualized percentiles at baseline (UKB dataset). Observed change from baseline to follow-up (x axis), and predicted rate of change, as estimated from cross-sectional baseline data (using individualized percentiles at baseline). **Abbreviations:** gray matter volume (GMV); cortical thickness (CTh); surface area (SA); fractional anisotropy (FA); Cross (cross-sectional).

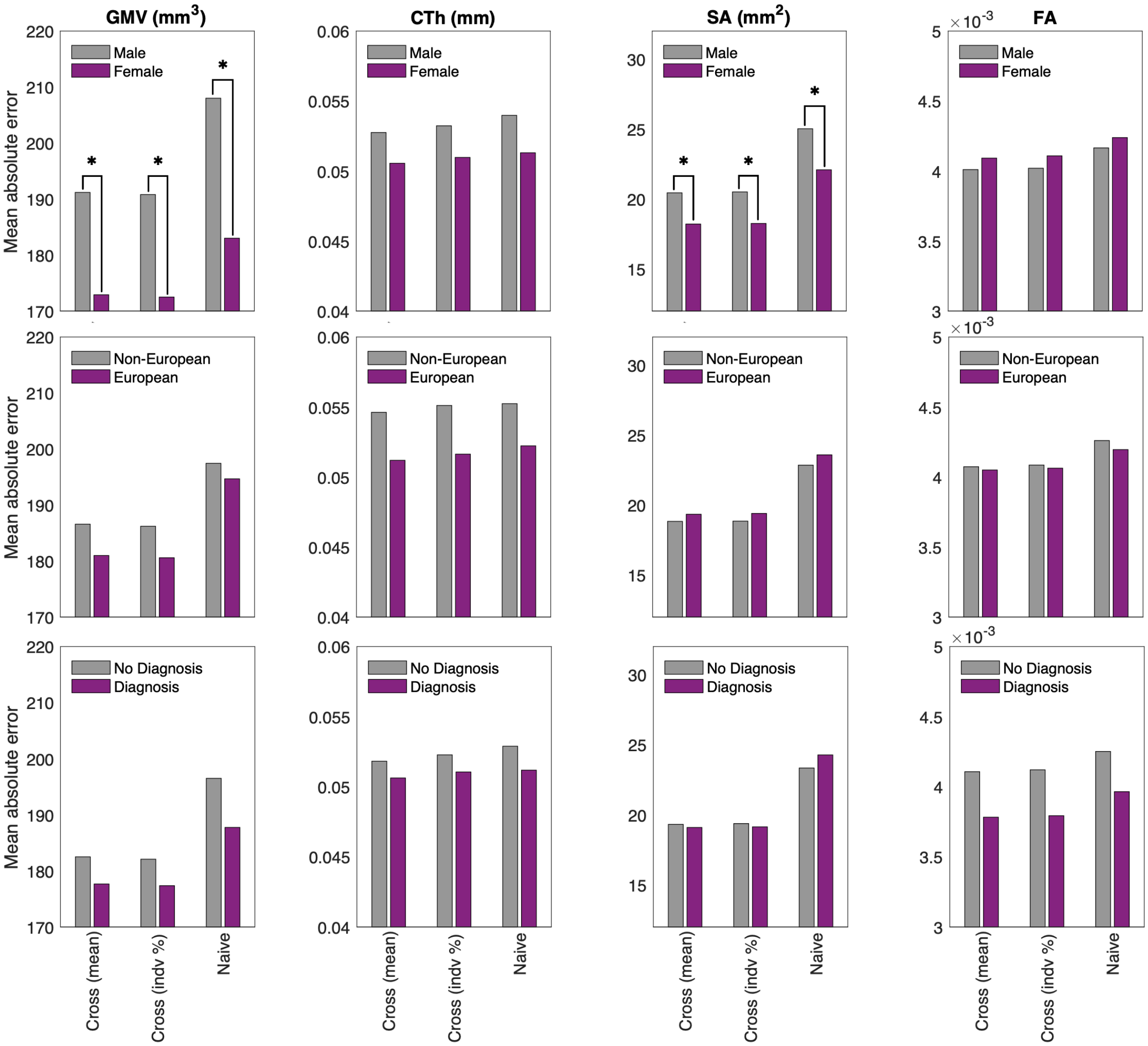

Fig. S6. The influence of person-specific factors on predicting individualized trajectories (UKB dataset). Absolute error from predicting rate of change with cross-sectional models based on the 50-th percentile (mean) and individualized percentiles at baseline (indv %), and naïve models (i.e., follow-up phenotype values are equal to baseline phenotype values). Bars denote group means and asterisks denote statistically significant comparisons after correction with the false discovery rate (*FDR*p<0.01). The following groups were compared on variability in MAE: i) females and males (top row), ii) European ancestry and non-European ancestry (middle row) and iii) with and without a medical/psychiatric/neurologic diagnosis. **Abbreviations:** gray matter volume (GMV); cortical thickness (CTh); surface area (SA); fractional anisotropy (FA); Cross (cross-sectional).

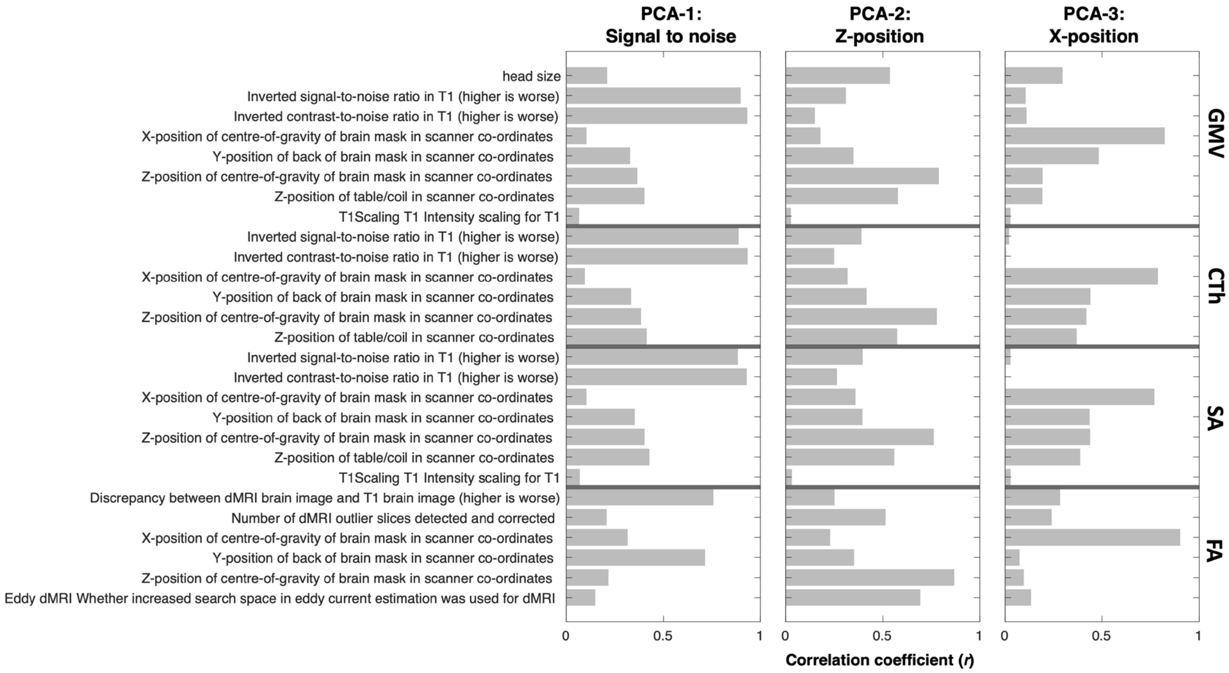

Fig. S7. Principal component analysis of neuroimaging confounds (UKB dataset). Plots display absolute correlation coefficients (r-values) between each neuroimaging confound with the first 3 components derived from principal component analysis (PCA). Components were defined (in the main text) based on the neuroimaging confound displaying the strongest relationship to corresponding components (absolute r-value). **Abbreviations:** gray matter volume (GMV); cortical thickness (CTh); surface area (SA); fractional anisotropy (FA).

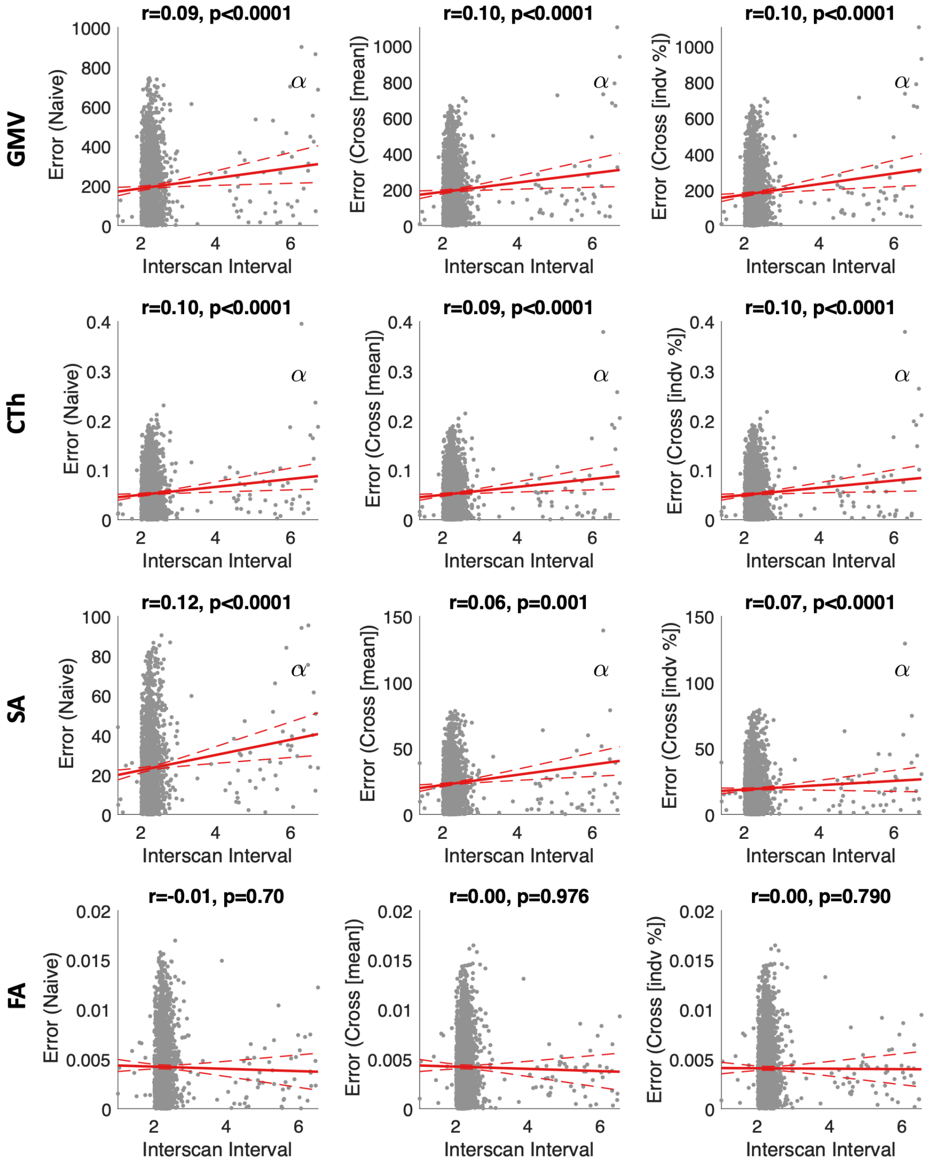

Fig. S8. Prediction error associations with interscan time interval (UKB dataset). Scatter plots present correlations between interscan time interval in years (x-axis) and the mean absolute error (y-axis). Alpha denotes significance after multiple comparison correction (FDRp<0.01). **Abbreviations:** gray matter volume (GMV); cortical thickness (CTh); surface area (SA); fractional anisotropy (FA); Cross (cross-sectional); indv (individualized).

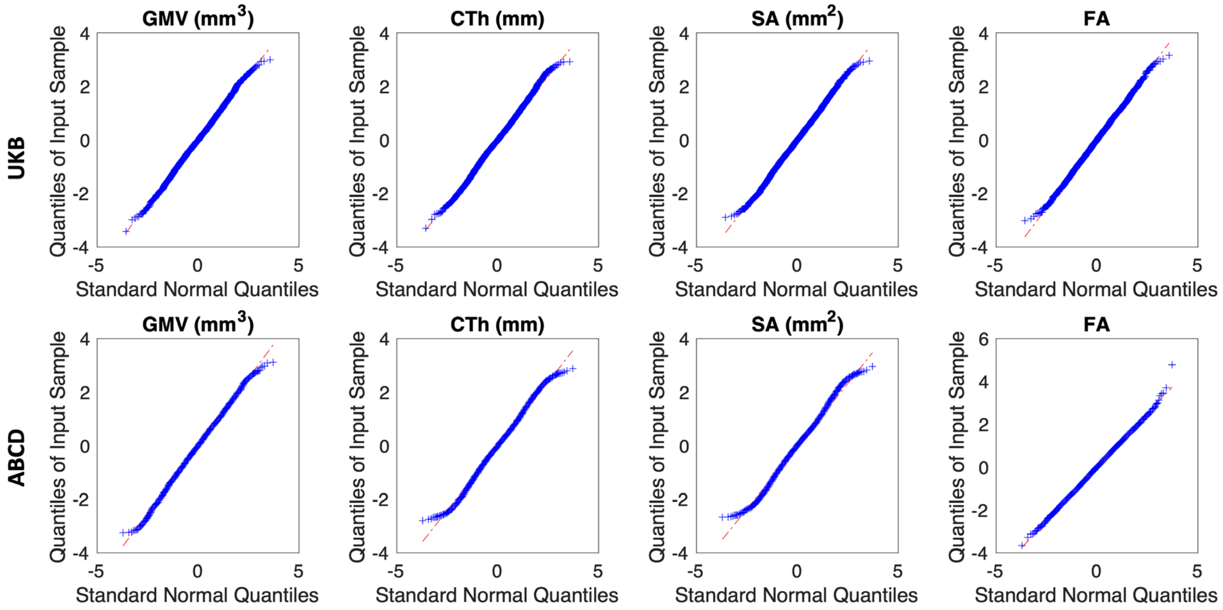

Fig. S9. QQplots visualizing GAMLSS model fits. Data (blue) versus standard normal. To assess model fit, the quantiles of the fitted distribution for each individual measurement were mapped to z-scores of a standard normal distribution and Q-Q plots were used to visually assess that the z-score normality. The Kolmogorov–Smirnov (KS) test was used to confirm the normality of z-scores for all phenotypes (refer to Table S4 KS results). Additionally, goodness of model fit was determined by minimizing the Akaike Information Criterion (AIC) index. **Abbreviations:** gray matter volume (GMV); cortical thickness (CTh); surface area (SA); fractional anisotropy (FA).

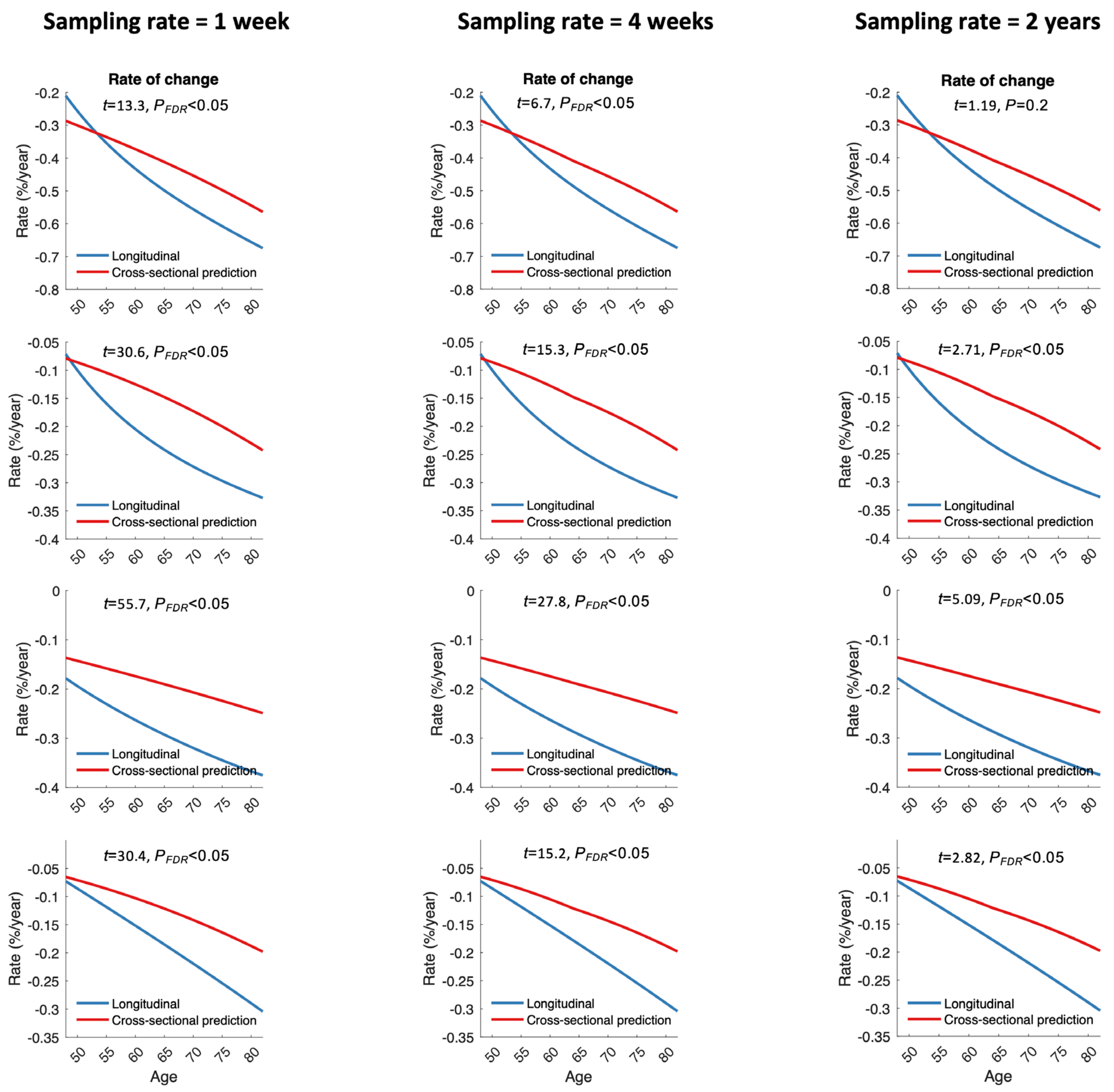

Fig. S10. Discrepancies between cross-sectional and longitudinal estimates using different sampling rates. Using alternative sampling rates/temporal resolutions across the age range did not induce an appreciable impact on the results, and in turn, conclusions drawn. Here, the age range was uniformly sampled at a resolution of 1 week (**Left panel**), 4 weeks (**Middle panel**) and 2 years (**Right panel**). As shown, rates of age-related change were lower for cross-sectional estimates relative to longitudinal estimates across all four phenotypes. However, this effect was not significant in terms of CTh using a sampling resolution of 2 years, which is likely due to the low number of datapoints (n=20) used for statistical testing. Note that these results are based on GAMLSS models computed across the entire sample, whereas the main results (see Figure 2) were derived from bootstrapping. **Abbreviations:** gray matter volume (GMV); cortical thickness (CTh); surface area (SA); fractional anisotropy (FA).

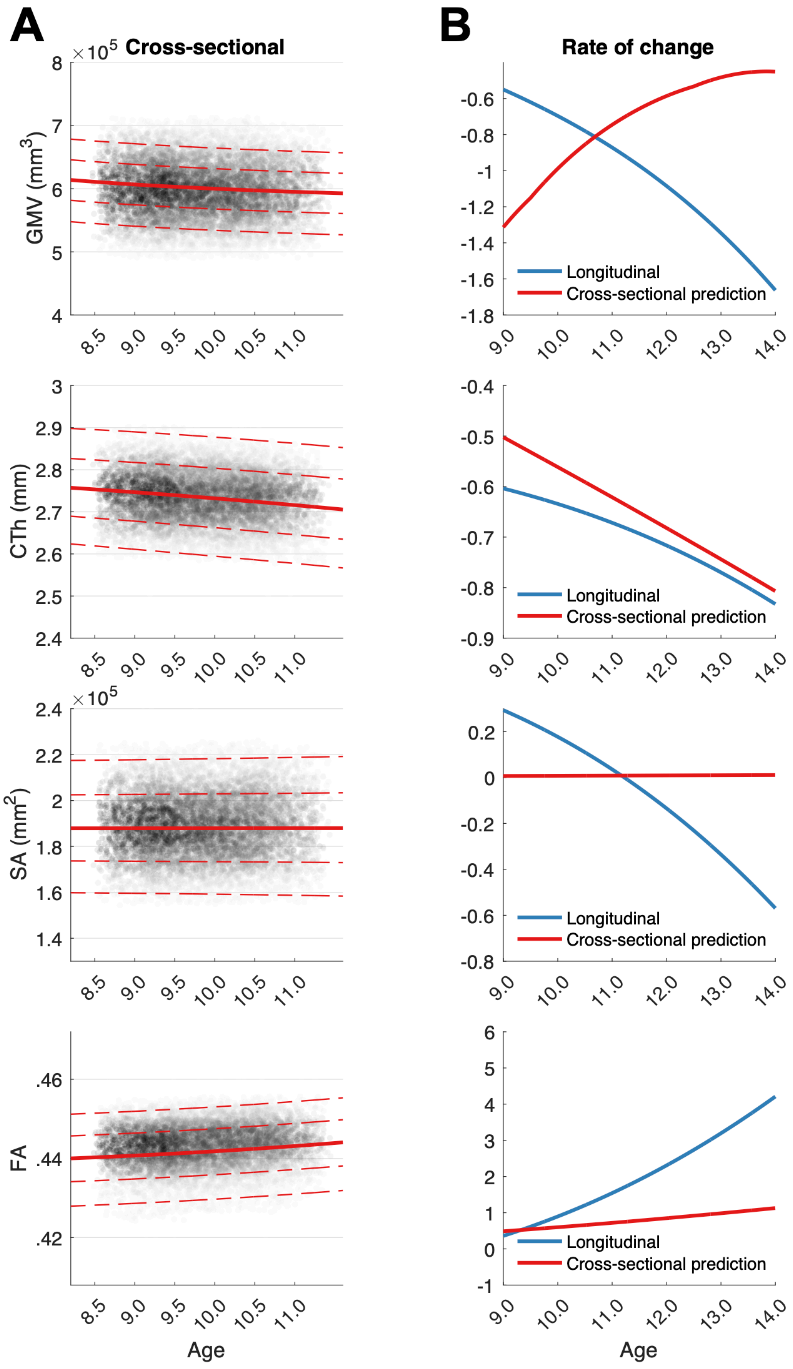

Fig. S11. Normative models of brain development (ABCD dataset). Normative centile reference ranges for **(A)** cross-sectionally measured whole-brain GMV, CTh, SA and FA and **(B)** rates of change for each phenotype. Rates of change were estimated: i) directly from longitudinally measured phenotypes (blue); and ii) by differentiating the median centile curves (red). **Abbreviations:** gray matter volume (GMV); cortical thickness (CTh); surface area (SA); fractional anisotropy (FA).

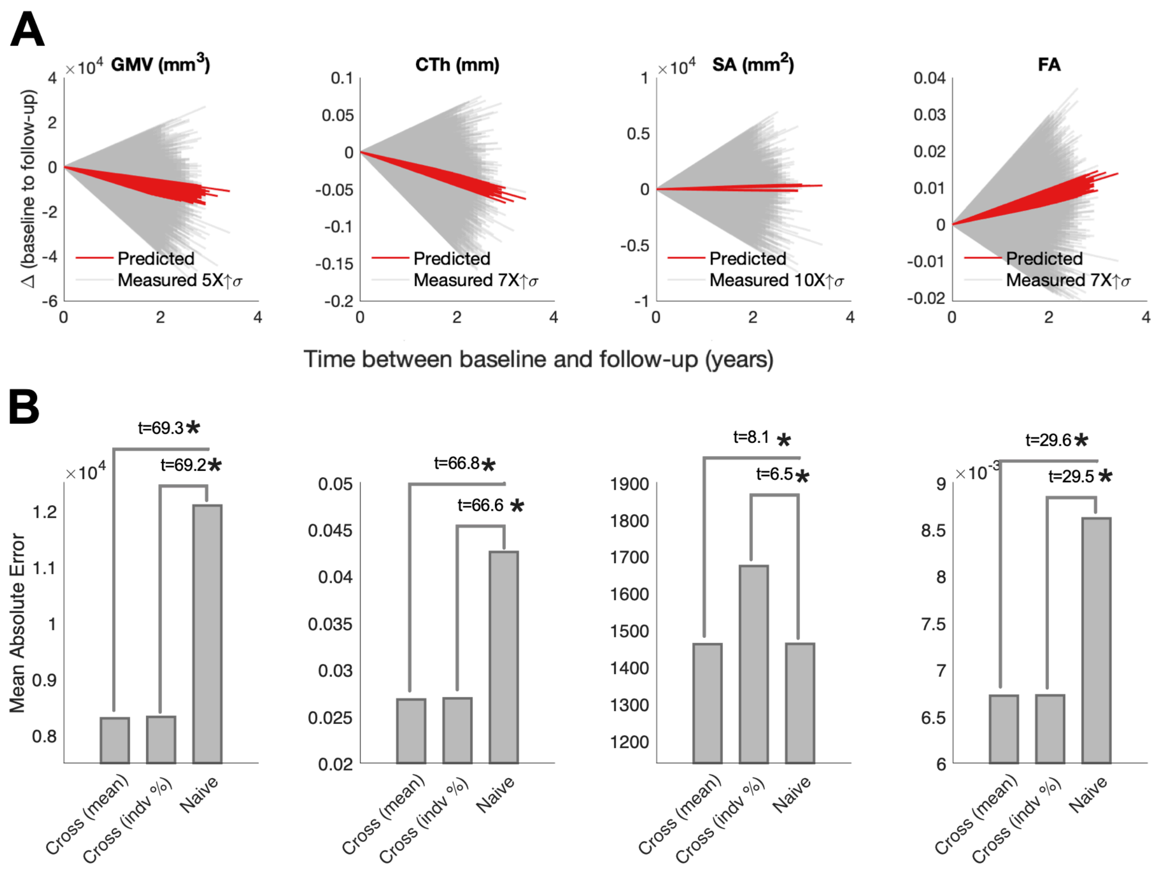

Fig. S12. Predicting individualized trajectories from group-level age trends (ABCD dataset). (A) Observed change from baseline to follow-up (x axis), and predicted rate of change, as estimated from cross-sectional (cross) baseline data (using the 50-th for all individuals). **(B)** Mean absolute error in predicting rate of change with cross-sectional models based on the 50-th percentile (mean) and individualized percentiles at baseline (indv %), and naïve models (i.e., follow-up phenotype values are equal to baseline phenotype values). Bars denote between-group comparisons and asterisks denote significance (P_{FDR}<0.01). **Abbreviations:** gray matter volume (GMV); cortical thickness (CTh); surface area (SA); fractional anisotropy (FA); Cross (cross-sectional); indv (individualized).

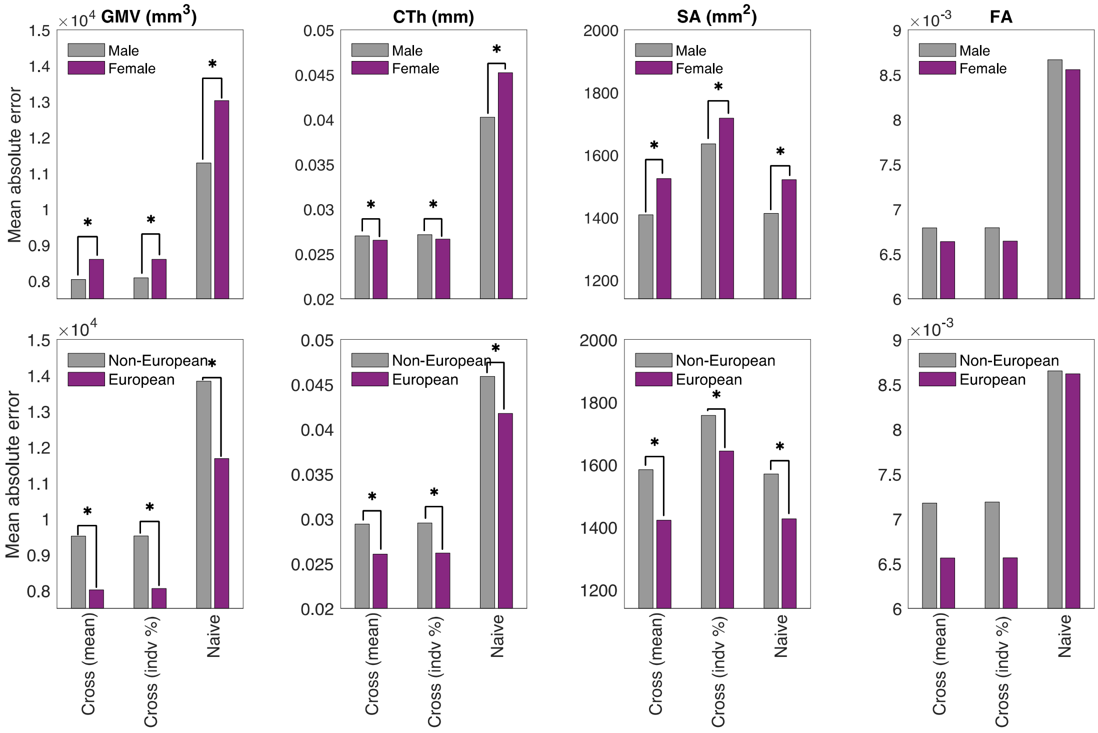

Fig. S13. The influence of person-specific factors on predicting individualized trajectories (ABCD dataset). Absolute error from predicting rate of change with cross-sectional models based on the 50-th percentile (mean) and individualized percentiles at baseline (indv %), and naïve models (i.e., follow-up phenotype values are equal to baseline phenotype values). Bars denote group means and asterisks denote statistically significant comparisons after correction with the false discovery rate (*FDR*p<0.01). The following groups were compared on variability in MAE: i) females and males (top row) and ii) European ancestry and non-European ancestry (bottom row). **Abbreviations:** gray matter volume (GMV); cortical thickness (CTh); surface area (SA); fractional anisotropy (FA); Cross (cross-sectional).

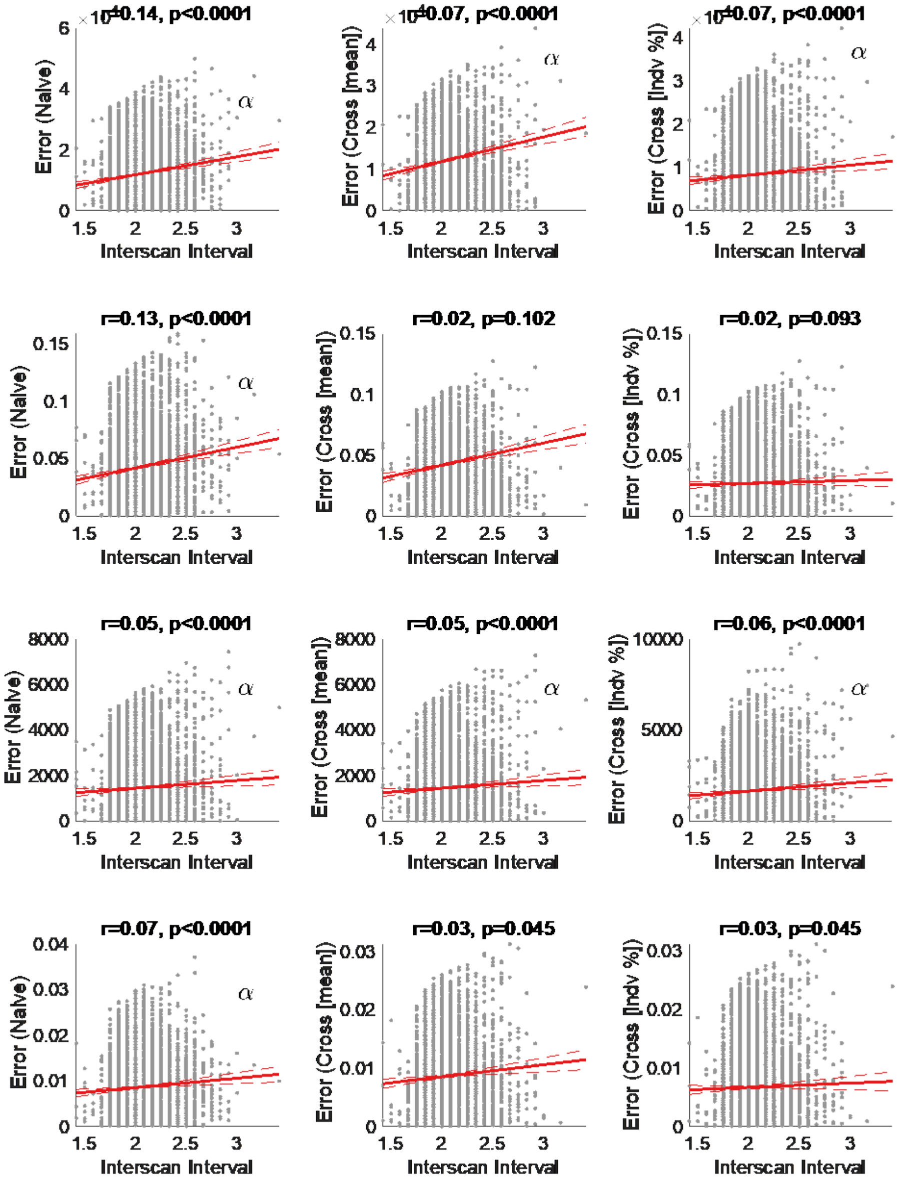

Fig. S14. Prediction error associations with interscan time interval (ABCD dataset). Scatter plots present correlations between interscan time interval in years (x-axis) and the mean absolute error (y-axis). Alpha denotes significance after multiple comparison correction (FDRp<0.01). **Abbreviations:** gray matter volume (GMV); cortical thickness (CTh); surface area (SA); fractional anisotropy (FA); Cross (cross-sectional); indv (individualized).

Table S1. Demographics.

UKB	GMV n=2787				CTh n=2763				SA n=2752				FA n=2832			
	**Mean**	**SD**	**Min**	**Max**	**Mean**	**SD**	**Min**	**Max**	**Mean**	**SD**	**Min**	**Max**	**Mean**	**SD**	**Min**	**Max**
Age at first MRI	62.9	7.2	47.0	80.3	62.9	7.3	47.0	80.3	62.8	7.2	47.0	80.3	62.9	7.3	47.0	80.3
Age at repeat MRI	65.2	7.2	49.3	82.6	65.2	7.2	49.3	82.6	65.1	7.2	49.3	82.6	65.2	7.2	49.3	82.6
Interscan Interval (years)	2.31	0.44	1.39	6.73	2.30	0.44	1.39	6.73	2.31	0.45	1.39	6.73	2.31	0.44	1.00	6.53
	**n**	**%**			**n**	**%**			**n**	**%**			**n**	**%**		
Sex (males)	1340	48			1341	49			1319	48			1353	48		
Genetic Grouping (white)	2441	88			2420	88			2414	88			2493	88		
No diagnosis	470	17			468	17			460	17			473	17		
**ABCD**	**GMV** n=7480				**CTh** n=7153				**SA** n=7069				**FA** n=6537			
	**Mean**	**SD**	**Min**	**Max**	**Mean**	**SD**	**Min**	**Max**	**Mean**	**SD**	**Min**	**Max**	**Mean**	**SD**	**Min**	**Max**
Age at first MRI	9.9	0.6	8.9	11.1	9.9	0.6	8.9	11.1	9.9	0.6	8.9	11.1	9.9	0.6	8.9	11.1
Age at repeat MRI	11.9	0.6	10.6	13.8	11.9	0.6	10.6	13.8	11.9	0.6	10.6	13.8	11.9	0.6	10.6	13.8
Interscan Interval (years)	2.0	0.2	1.4	3.4	2.0	0.2	1.4	3.4	2.0	0.2	1.4	3.4	2.0	0.2	1.4	3.4
	**n**	**%**			**n**	**%**			**n**	**%**			**n**	**%**		
Sex (males)	4032	54			3832	54			3811	54			3517	54		
**Ethnicity:**																
White	5759	77			5534	77			5437	77			5082	78		
Black/African American	1096	15			1019	14			1054	15			933	14		
American Indian and/or Alaska Native	46	1			44	1			41	1			39	1		
Asian	159	2			155	2			147	2			128	2		
Native Hawaiian and/or other Pacific Islander	16	0			15	0			15	0			14	0		
Missing	404	5			386	5			375	5			341	5		

Demographics for all subjects with longitudinal data for each modality (after removing outliers from imaging data).

Table S2. MAE associations with individual neuroimaging confounds.

UKB	MAE Variance Explained		
**GMV**	Naive	Cross (Mean)	Cross (indv %)
Total intracranial volume	0.00244	0.00185	0.00170
Inverted signal-to-noise ratio in T1 (higher is worse)	0.00000	0.00029	0.00030
Inverted contrast-to-noise ratio in T1 (higher is worse)	0.00006	0.00016	0.00016
X-position of centre-of-gravity of brain mask in scanner co-ordinates	0.00000	0.00070	0.00072
Y-position of back of brain mask in scanner co-ordinates	0.00251	0.00013	0.00014
Z-position of centre-of-gravity of brain mask in scanner co-ordinates	0.00464	0.00002	0.00003
Z-position of table/coil in scanner co-ordinates	0.00067	0.00136	0.00150
T1Scaling T1 Intensity scaling for T1	0.00172	0.00560	0.00548
**CTh**			
Inverted signal-to-noise ratio in T1 (higher is worse)	0.00028	0.00021	0.00024
Inverted contrast-to-noise ratio in T1 (higher is worse)	0.00006	0.00002	0.00003
X-position of centre-of-gravity of brain mask in scanner co-ordinates	0.00008	0.00020	0.00019
Y-position of back of brain mask in scanner co-ordinates	0.00005	0.00002	0.00001
Z-position of centre-of-gravity of brain mask in scanner co-ordinates	0.00139	0.00106	0.00094
Z-position of table/coil in scanner co-ordinates	0.00000	0.00001	0.00003
**SA**			
Inverted signal-to-noise ratio in T1 (higher is worse)	0.00229	0.00760	0.00768
Inverted contrast-to-noise ratio in T1 (higher is worse)	0.00024	0.00183	0.00183
X-position of centre-of-gravity of brain mask in scanner co-ordinates	0.00467	0.00131	0.00147
Y-position of back of brain mask in scanner co-ordinates	0.00828	0.00051	0.00047
Z-position of centre-of-gravity of brain mask in scanner co-ordinates	0.02764	0.00541	0.00601
Z-position of table/coil in scanner co-ordinates	0.00370	0.00090	0.00080
T1Scaling T1 Intensity scaling for T1	0.00002	0.00008	0.00015
**FA**			
Discrepancy between dMRI brain image and T1 brain image (higher is worse)	0.00008	0.00004	0.00005
Number of dMRI outlier slices detected and corrected	0.00009	0.00025	0.00023
X-position of centre-of-gravity of brain mask in scanner co-ordinates	0.00004	0.00124	0.00120
Y-position of back of brain mask in scanner co-ordinates	0.00121	0.00015	0.00014
Z-position of centre-of-gravity of brain mask in scanner co-ordinates	0.01039	0.00457	0.00463
Eddy dMRI: Whether increased search space in eddy current estimation was used for dMRI	0.00366	0.00011	0.00010

ABCD	MAE Variance Explained		
**GMV**	Naive	Cross (Mean)	Cross (indv %)
Total intracranial volume	0.00086	0.00014	0.00006
Number of topology defects for left and right hemispheres before topology correction	0.00025	0.02589	0.02531
Normalized score indicating displacement and rotation of T2w volume relative to T1w volume	0.00013	0.00064	0.00067
Normalized score indicating stretching of T2w volume relative to T1w volume	0.00092	0.00024	0.00027
**CTh**			
Number of topology defects for left and right hemispheres before topology correction	0.00489	0.03686	0.03704
Normalized score indicating displacement and rotation of T2w volume relative to T1w volume	0.00019	0.00077	0.00079
Normalized score indicating stretching of T2w volume relative to T1w volume	0.00160	0.00292	0.00297
**SA**			
Number of topology defects for left and right hemispheres before topology correction	0.04361	0.04492	0.03324
Normalized score indicating displacement and rotation of T2w volume relative to T1w volume	0.00046	0.00055	0.00026
Normalized score indicating stretching of T2w volume relative to T1w volume	0.00341	0.00402	0.00196
**FA**			
Maximum dorsal cutoff score in dMRI FM images	0.00053	0.00045	0.00049
Maximum ventral cutoff score in dMRI FM images	0.00238	0.00457	0.00453
Scans available for dMRI B0 unwarp	0.00178	0.00207	0.00212
Phase-encode polarity difference score for dMRI fieldmap	0.00048	0.04926	0.04913
Root mean square of estimated B0 displacements in phase-encode direction for dMRI fieldmap	0.00286	0.06003	0.05998
Normalized score indicating displacement and rotation of dMRI fieldmap relative to T1w volume	0.00083	0.03401	0.03405
Normalized score indicating stretching of dMRI fieldmap relative to T1w volume	0.00251	0.02361	0.02364

P_{FDR} <0.05 demarcated in blue

Table S3. Sample filtering.

**UKB**	**GMV**		**CTh**		**SA**		**FA**	
Total n with IDP at 2 TPs	2858		2858		2858		2890	
	**Removed**		**Removed**		**Removed**		**Removed**	
	**n**	**%**	**n**	**%**	**n**	**%**	**n**	**%**
Outlier at baseline	17	1	22	1	20	1	12	0
Outlier at follow-up	8	0	23	1	3	0	23	1
Outlier on Rate of change	46	2	50	2	83	3	23	1
Total n excluded	71	3	95	3	106	4	58	2
**Final sample**	**2787**		**2763**		**2752**		**2832**	
**ABCD**	**GMV**		**CTh**		**SA**		**FA**	
Total n with IDP at 2 TPs	7736		7736		7736		7156	
	**Removed**		**Removed**		**Removed**		**Removed**	
	**n**	**%**	**n**	**%**	**n**	**%**	**n**	**%**
Missing Site	1	<1	1	<1	1	<1		
Outlier at baseline	21	<1	80	1	22	<1	183	3
Outlier at follow-up	4	<1	58	<1	3	<1	79	1
Outlier on Rate of change	230	3	444	6	641	9	357	5
Total n excluded	256	3	583	8	667	9	619	9
**Final sample**	**7480**		**7153**		**7069**		**6537**	

Outliers were defined as values more than three scaled median absolute deviations (MAD) away from the median

Table S4. Kolmogorov–Smirnov (KS) Results.

		GMV	CTh	SA	FA
**UKB**	KS Statistic	0.012	0.011	0.013	0.010
	KS P-value	0.825	0.876	0.748	0.941
**ABCD**	KS Statistic	0.007	0.007	0.006	0.007
	KS P-value	0.850	0.850	0.980	0.930

SI References

1. C. Sudlow *et al.*, UK biobank: an open access resource for identifying the causes of a wide range of complex diseases of middle and old age. *PLoS medicine* **12**, e1001779 (2015).
2. T. L. Jernigan, S. A. Brown, G. J. Dowling, The adolescent brain cognitive development study. *Journal of research on adolescence: the official journal of the Society for Research on Adolescence* **28**, 154 (2018).
3. K. L. Miller *et al.*, Multimodal population brain imaging in the UK Biobank prospective epidemiological study. *Nature neuroscience* **19**, 1523-1536 (2016).
4. F. Alfaro-Almagro *et al.*, Image processing and Quality Control for the first 10,000 brain imaging datasets from UK Biobank. *NeuroImage* **166**, 400-424 (2018).
5. B. Fischl, FreeSurfer. *NeuroImage* **62** (2012).
6. M. Jenkinson, C. F. Beckmann, T. E. Behrens, M. W. Woolrich, S. M. Smith, Fsl. *NeuroImage* **62**, 782-790 (2012).
7. S. M. Smith *et al.*, Advances in functional and structural MR image analysis and implementation as FSL. *NeuroImage* **23, Supplement 1**, S208-S219 (2004).
8. S. Smith, F. A. Almagro, K. Miller (UK Biobank Brain Imaging Documentation.
9. A. M. Dale, B. Fischl, M. I. Sereno, Cortical surface-based analysis: I. Segmentation and surface reconstruction. *NeuroImage* **9**, 179-194 (1999).
10. Anonymous (AutoPtx.
11. B. Casey *et al.*, The adolescent brain cognitive development (ABCD) study: imaging acquisition across 21 sites. *Dev Cogn Neurosci* **32**, 43-54 (2018).
12. D. J. Hagler *et al.*, Image processing and analysis methods for the Adolescent Brain Cognitive Development Study. *NeuroImage* **202**, 116091 (2019).
13. R. W. Cox, AFNI: software for analysis and visualization of functional magnetic resonance neuroimages. *Computers and Biomedical research* **29**, 162-173 (1996).
14. D. o. Health (2016) Alcohol guidelines review–Report from the Guidelines development group to the UK Chief Medical Officers. (Department of Health London).
15. E. Inan-Eroglu *et al.*, Is There a Link between Different Types of Alcoholic Drinks and Obesity? An Analysis of 280,183 UK Biobank Participants. *International journal of environmental research and public health* **17**, 5178 (2020).
16. K. Perreault *et al.*, Does physical activity moderate the association between alcohol drinking and all-cause, cancer and cardiovascular diseases mortality? A pooled analysis of eight British population cohorts. *British journal of sports medicine* **51**, 651-657 (2017).
17. I. Lourida *et al.*, Association of Lifestyle and Genetic Risk With Incidence of Dementia. *JAMA* **322**, 430-437 (2019).
18. D. Mozaffarian, Dietary and policy priorities for cardiovascular disease, diabetes, and obesity: a comprehensive review. *Circulation* **133**, 187-225 (2016).
19. K. E. Bradbury, H. J. Young, W. Guo, T. J. Key, Dietary assessment in UK Biobank: an evaluation of the performance of the touchscreen dietary questionnaire. *Journal of nutritional science* **7** (2018).
20. M. o. A. Fisheries, Food (1993) Food portion sizes. (HMSO London, United Kingdom).
21. M. Roe, H. Pinchen, S. Church, P. Finglas, McC ance and Widdowson's the composition of foods seventh summary edition and updated composition of foods integrated dataset. *Nutrition bulletin* **40**, 36-39 (2015).
22. Anonymous (The IPAQ Group, IPAQ scoring protocol - International Physical Activity Questionnaire. Available at: https://sites.google.com/site/theipaq/scoring-protocol.

23. J. C. Gray *et al.*, Associations of cigarette smoking with gray and white matter in the UK Biobank. *Neuropsychopharmacology : official publication of the American College of Neuropsychopharmacology* **45**, 1215-1222 (2020).

24. R. A. Rigby, D. M. Stasinopoulos, Generalized additive models for location, scale and shape. *Journal of the Royal Statistical Society: Series C (Applied Statistics)* **54**, 507-554 (2005).

25. M. D. Stasinopoulos, R. A. Rigby, G. Z. Heller, V. Voudouris, F. De Bastiani, *Flexible regression and smoothing: using GAMLSS in R* (CRC Press, 2017).

26. R. A. Rigby, D. M. Stasinopoulos, Using the Box-Cox t distribution in GAMLSS to model skewness and kurtosis. *Statistical Modelling* **6**, 209-229 (2006).

27. M. Taquet *et al.*, A structural brain network of genetic vulnerability to psychiatric illness. *Molecular psychiatry*, 1-12 (2020).

28. C. Destrieux, B. Fischl, A. Dale, E. Halgren, Automatic parcellation of human cortical gyri and sulci using standard anatomical nomenclature. *NeuroImage* **53**, 1-15 (2010).

29. S. Mori, S. Wakana, P. C. Van Zijl, L. Nagae-Poetscher, *MRI atlas of human white matter* (Elsevier, Amsterdam, 2005).
